# Exercise Attenuates the Transition from Fatty Liver to Steatohepatitis and Reduces Tumor Formation in Mice

**DOI:** 10.3390/cancers12061407

**Published:** 2020-05-29

**Authors:** Maria Guarino, Pavitra Kumar, Andrea Felser, Luigi M. Terracciano, Sergi Guixé-Muntet, Bostjan Humar, Michelangelo Foti, Jean-Marc Nuoffer, Marie V. St-Pierre, Jean-François Dufour

**Affiliations:** 1 Hepatology, Department for BioMedical Research, University of Bern, 3008 Bern, Switzerland; maria.guarino86@gmail.com (M.G.); pavitra.kumar@dbmr.unibe.ch (P.K.); sergi.guixe@dbmr.unibe.ch (S.G.-M.); marie.st-pierre@dbmr.unibe.ch (M.V.S.-P.); 2 Gastroenterology, Department of Clinical Medicine and Surgery, University of Naples “Federico II”, 80131 Naples, Italy; 3 University Institute of Clinical Chemistry, Bern University Hospital, 3010 Bern, Switzerland; andrea.felser@insel.ch (A.F.); Jean-Marc.Nuoffer@insel.ch (J.-M.N.); 4 Institute of Pathology, University Hospital Basel, 4056 Basel, Switzerland; luigi.terracciano@usb.ch; 5 Laboratory of the Swiss Hepato-Pancreato-Biliary (HPB) and Transplantation Center, Department of Surgery, University Hospital Zürich, 8091 Zürich, Switzerland; bostjan.humar@usz.ch; 6 Department of Cell Physiology and Metabolism, University of Geneva, 1206 Geneva, Switzerland; michelangelo.foti@unige.ch; 7 University Clinic of Visceral Surgery and Medicine, Inselspital Bern, 3010 Bern, Switzerland

**Keywords:** exercise, ER stress, non-alcoholic fatty liver disease (NAFLD), non-alcoholic steatohepatitis (NASH), hepatocellular carcinoma (HCC), liver fibrosis, high-fat diet

## Abstract

Non-alcoholic fatty liver disease (NAFLD) leads to steatohepatitis (NASH), fibrosis, and hepatocellular carcinoma. For sedentary patients, lifestyle interventions combining exercise and dietary changes are a cornerstone of treatment. However, the benefit of exercise alone when dietary changes have failed is uncertain. We query whether exercise alone arrests the progression of NASH and tumorigenesis in a choline-deficient, high-fat diet (CD-HFD) murine model. Male C57Bl/6N mice received a control diet or CD-HFD for 12 weeks. CD-HFD mice were randomized further for 8 weeks of sedentariness (SED) or treadmill exercise (EXE). CD-HFD for 12 weeks produced NAFL. After 20 weeks, SED mice developed NASH and hepatic adenomas. Exercise attenuated the progression to NASH. EXE livers showed lower triglycerides and tumor necrosis factor-α expression, less fibrosis, less ballooning, and a lower NAFLD activity score than did SED livers. Plasma transaminases and triglycerides were lower. Exercise activated AMP-activated protein kinase (AMPK) with inhibition of mTORC1 and decreased S6 phosphorylation, reducing hepatocellular adenoma. Exercise activated autophagy with increased LC3-II/LC3-I and mitochondrial recruitment of phosphorylated PTEN-induced kinase. Therefore, exercise attenuates the transition from NAFL to NASH, improves biochemical and histological parameters of NAFLD, and impedes the progression of fibrosis and tumorigenesis associated with enhanced activation of AMPK signaling and favors liver autophagy. Our work supports the benefits of exercise independently of dietary changes.

## 1. Introduction

Non-alcoholic fatty liver disease (NAFLD) affects nearly 25% of the world population [1], and its economic burden on society is projected to increase along with its co-morbidities [2]. Clinically, NAFLD covers a spectrum of conditions ranging from non-alcoholic fatty liver (NAFL) to decompensated liver cirrhosis, which can progress to hepatocellular carcinoma (HCC). NAFL is defined by steatosis with or without inflammation but without ballooning injury. In contrast, non-alcoholic steatohepatitis (NASH) is defined by the presence of ballooning injury with inflammation in addition to steatosis [3]. The transition from fatty liver to NASH is an important milestone in the evolution of the disease. In a prospective study from the NASH clinical research network, changes in the NASH activity score, which includes ballooning injury as a marker, were associated with concordant changes in fibrosis [4]. In turn, the degree of hepatic fibrosis was related to both liver-related mortality of NAFLD and to the overall mortality [5]. Currently, no pharmacological treatments can arrest the progression of NAFL to NASH or reverse NASH once it is established, although several experimental drugs are being tested [6]. 

NAFLD in general and NASH in particular are associated with high caloric, high-fat diet, and sedentariness. Hence, NAFLD is linked to a metabolic overload of the liver. Consequently, clinical trials proposing weight loss and other lifestyle interventions as therapeutic measures have enrolled patients with NASH and fibrosis and sought to demonstrate the resolution of NASH and the reversal of fibrosis. In general, weight loss is positively correlated with an improvement in histologic features of NASH, but for a resolution of NASH and regression of fibrosis, weight loss must be superior to 10% [7,8]. One specific lifestyle intervention documented to improve the hepatic metabolic situation is regular physical activity [9]. In fact, physical activity reduces steatosis in patients with NAFLD, even when it is not associated with weight loss [10,11]. Moreover, resistance exercise in sedentary adults improves steatosis without an impact on body weight [12]. Nonetheless, the interpretation of these clinical studies is confounded when dietary and exercise interventions cannot be dissociated. Whether selective intervention of an exercise regime in patients who maintain their high-fat diet is beneficial and whether exercise alone can impede the transition from NAFL to NASH and thus prevent the evolution of fibrosis are uncertain. In addition, it is also unclear which signaling pathways are operative in transducing the beneficial effects of exercise when the NASH-inducing diet remains in place.

We previously reported that in a genetic PTEN knockout mouse model of NASH, regular physical activity decreases the incidence of HCC but without a change in the NAFLD activity score (NAS) score [13]. We also reported that in a rat model of orthotopic syngeneic tumor implantation, regular physical activity downregulated the expression of hepatic genes associated with the development of HCC [14]. However, it remains unknown whether an exercise regimen can intervene at all points, or only at specific points, along the continuum from NAFL to NASH to fibrosis and HCC and change the course of the disease, especially within the context of continued nutritional overload. To explore the selective benefits of physical activity in NAFLD triggered by a high-fat diet and to elucidate its mechanism, we queried whether daily exercise alone impedes the transition from NAFL to NASH and impairs the progression of fibrosis. We chose a choline-deficient, high-fat diet mouse model that displays a progressive phenotype of NAFL to NASH to hepatic fibrosis and, eventually, HCC and compared the disease outcome in sedentary and exercised mice [15,16].

## 2. Results

### 2.1. Exercise Attenuates the Transition from NAFL to NASH

**Figure 1:**
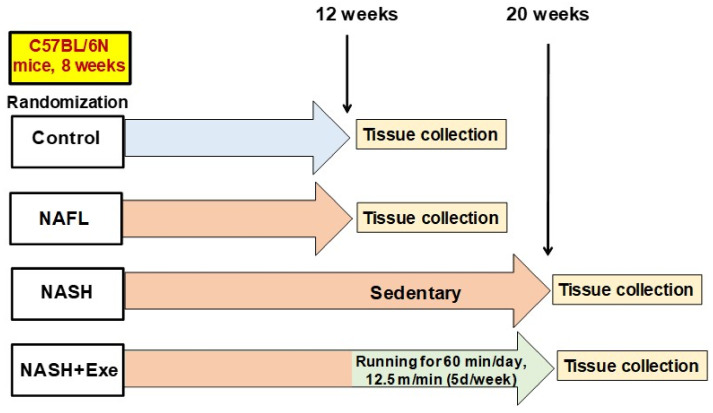
Schematic outline of the study design. Male C57Bl/6N mice were randomized to one of four groups: (1) the control group (*n* = 11) was fed a standard diet and tissues were collected after 12 weeks; (2) the non-alcoholic fatty liver (NAFL) group (*n* = 11) was fed a choline-deficient high-fat (CD-HFD) diet for 12 weeks before tissue collection; (3) the non-alcoholic steatohepatitis (NASH) group (*n* = 11) received a CD-HFD for 20 weeks and remained sedentary before tissue collection; (4) the NASH + exercise (EXE) group (*n* = 11) received a CD-HFD for 20 weeks but with treadmill running at 12.5 m/min imposed from weeks 12 to 20.

**Figure 2:**
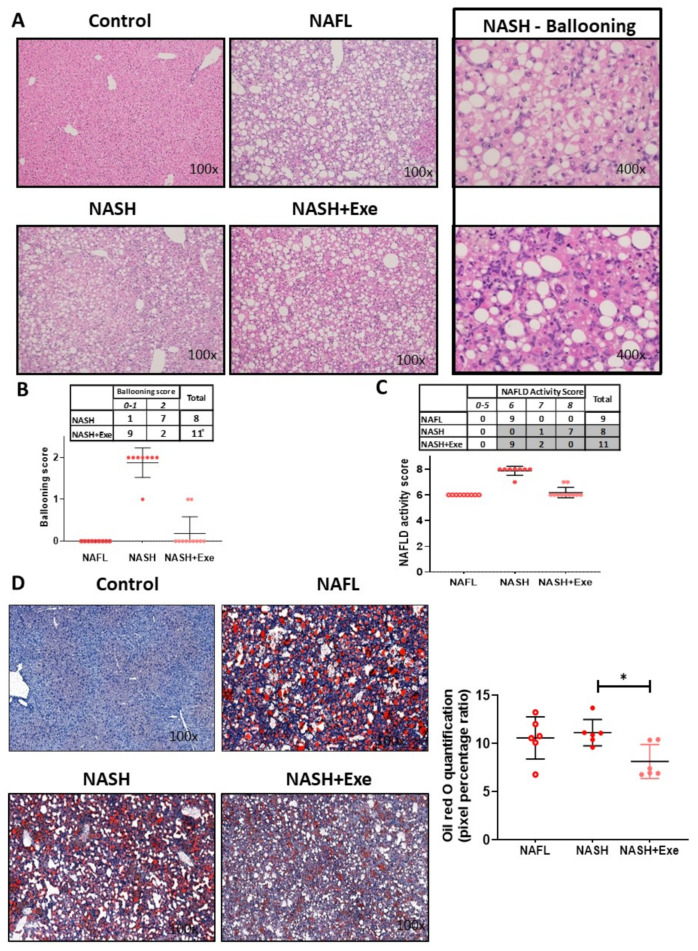
Effect of exercise on liver histology in mice fed a choline-deficient high-fat diet (CD-HFD). (**A**) Microscopy of hematoxylin and eosin (H&E)-stained liver sections showing diffuse macrovesicular steatosis in the NAFL, NASH and NASH + exercise (EXE) groups and the presence of ballooned hepatocytes only in the NASH sedentary group ). (**B**) Frequency table and dot plot comparing the ballooning score in the NASH sedentary and NASH+EXE groups. Ballooning was significantly lower in the NASH + EXE group (Fisher’s exact test, *p* = 0.005). (**C**) Frequency table and dot plot showing the NAFLD activity score in the NAFL, NASH sedentary, and NASH + EXE groups. The score was significantly lower in NASH + EXE than in the NASH sedentary group (Fisher’s exact test with Freeman–Halton extension, NASH vs. NASH + EXE, *p* < 0.0001). (**D**) Oil Red O staining comparing neutral lipid content of control, NAFL, NASH sedentary, and NASH + EXE livers. The quantification of lipid staining (right panel) was done with MetaMorph® analysis software. Lipid content was lower in the NASH + EXE livers (unpaired *t*-test; * *p <* 0.05*).*

We initially assessed the effect of CD-HFD and exercise (Figure 1) on liver lesions by grading the degree of steatosis. Compared to control mice, all CD-HFD-fed mice developed extensive steatosis (>90% steatosis, grade 3; Figure 2A). This macrovesicular steatosis was evident in the early 12-week treated CD-HFD group and in both the sedentary and exercise 20 week CD-HFD groups. Hepatocellular ballooning, a requisite histological feature for diagnosing NASH, was absent in 12-week CD-HFD mice. However, ballooning was present after 20 weeks of CD-HFD but was significantly attenuated by exercise. Ballooning was observed in all eight mice of the sedentary group (grade 2 in 90% of mice) predominantly in zone 3, whereas it was recorded in only 28% of the exercise group (Figure 2B). Neither Mallory–Denk bodies nor apoptotic bodies were detected in either group. Consistent with the development of NAFL and NASH, all CD-HFD fed mice showed hepatic lobular inflammation of grade 3. The NAS score increased significantly in the sedentary mice when compared to the exercised mice (Figure 2C). Oil Red O staining assessed the levels of triglycerides and other neutral lipids in the livers (Figure 2D). In both the 12-week and 20-week CD-HFD groups, the lipid staining was abundant. The signal was decreased in the exercised livers.

### 2.2. Exercise Halts the Progression of Fibrosis

**Figure 3:**
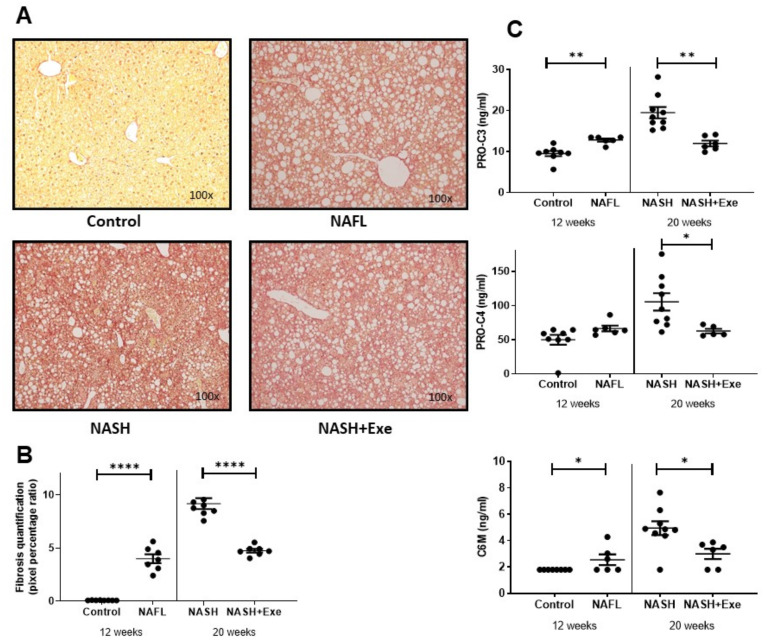
Effect of exercise on liver fibrosis. (**A**) Microscopy of Sirius Red-stained liver sections from C57BL/6N mice fed a control diet or a choline-deficient high-fat diet (CD-HFD). Fibrosis was absent in controls. Diffuse lobular pericellular fibrosis was present in the NAFL and NASH + exercise (EXE) groups but was highest in the NASH sedentary group. (**B**) Quantification of fibrosis shown in panel A. Images were quantified with the MetaMorph® analysis software. Fibrosis was higher in NAFL than in control groups and higher in NASH sedentary than in NASH + EXE group (unpaired *t*-test; ****p < 0.0001). (**C**) Fibrosis biomarkers in plasma. PRO-C3, PRO-C4, and C6M concentrations were measured in the plasma of mice from the control, NAFL, NASH, and NASH + EXE groups. PRO-C3 and C6M were significantly higher in NAFL than in controls and in NASH sedentary than in NASH+EXE (unpaired *t*-test; *p < 0.05; **p < 0.005).

To investigate the influence of exercise on liver fibrogenesis, we assessed total collagen in the liver and quantified circulating levels of three biomarkers, Pro-C3, Pro-C4, and C6M, in the plasma. After 12 weeks, NAFL mice displayed diffuse pericellular fibrosis but no portal fibrosis (Figure 3A). After 20 weeks, sedentary mice displayed even more fibrosis, but this change was halted by exercise (Figure 3B). In fact, the extent of fibrosis in the exercised mice was comparable to that observed in 12-week NAFL livers. Pro-C3, the proteolytic propeptide released during the formation of type III collagen, was significantly elevated after 12 weeks of CD-HFD, increased further after 20 weeks of CD-HFD in sedentary mice but this rise was halted in exercised mice (Figure 3C). Another biomarker, Pro-C4, which reflects the synthesis of the basement membrane collagen, type IV, was less affected by early CD-HFD. However, it increased after 20 weeks of CD-HFD in sedentary mice, a rise that was prevented by exercise. C6M, a degradation marker for type VI collagen, was slightly increased after 12 weeks of CD-HFD, rose further after 20 weeks of CD-HFD in sedentary mice, but this rise was prevented by exercise (Figure 3C). Thus, the changes in biomarkers were in keeping with the histological patterns observed.

### 2.3. Exercise Improves Biochemical Plasma Markers of NAFLD

**Figure 4:**
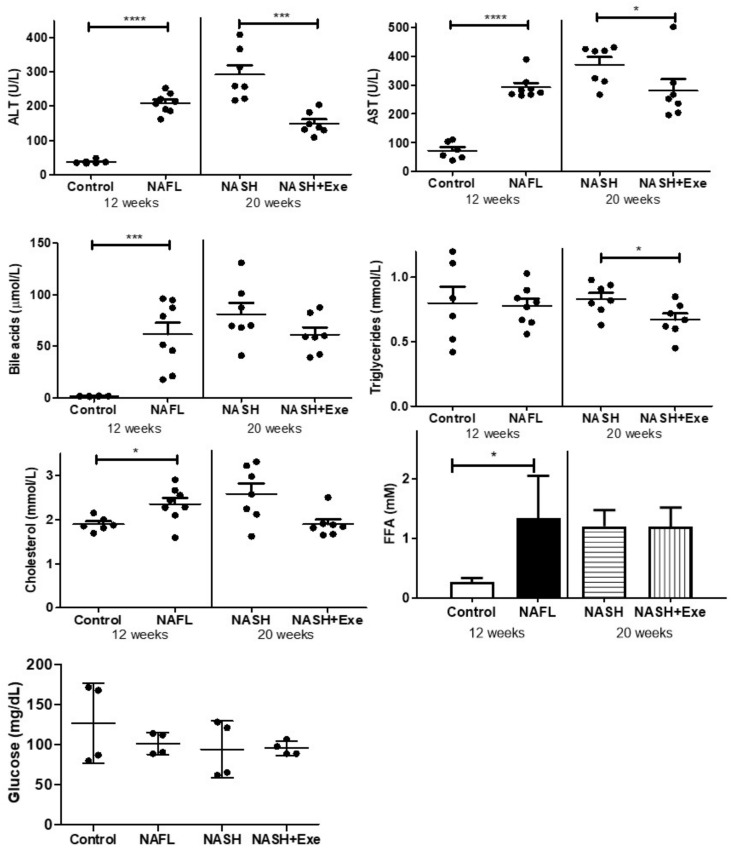
Effect of exercise on biochemical values in plasma. Plasma concentrations of alanine transaminase (ALT), aspartate transaminase (AST), total bile acids, triglycerides, cholesterol and free fatty acids (FFA), and fasting blood glucose were compared in mice fed a control diet or a choline-deficient high-fat diet (CD-HFD) for 12 weeks (NAFL), and in mice fed a CD-HFD for 20 weeks with (NASH + EXE) or without exercise (NASH). ALT and AST were higher in NAFL than in controls, and higher in NASH sedentary than in NASH + EXE (unpaired *t*-test; *p < 0.05; ***p < 0.001; ****p < 0.0001). Bile acids were elevated in NAFL vs. controls (*p* < 0.001). Triglycerides were lower in NASH + EXE than in NASH sedentary (*p* < 0.05). Cholesterol was higher in NAFL than in controls. FFA was higher in NAFL than in controls (unpaired *t*-test; *p* < 0.05).

Biochemical markers indicative of liver disease were measured in the plasma. Concentrations of both transaminases, ALT and AST, were increased at 12 weeks and further increased after 20 weeks of CD-HFD, but only in sedentary mice (Figure 4). Total bile acids increased after 12 weeks of CD-HFD, but the differences between sedentary and exercised mice at 20 weeks were not statistically significant. Exercise tended to lower plasma triglycerides. Cholesterol increased modestly after 12 weeks of CD-HFD and tended to be lower at 20 weeks in the exercised group. Free fatty acids (FFA) concentrations were increased after 12 weeks of CD-HFD, but the 20-week sedentary and exercise groups were not different. Fasting blood glucose was not different between the groups.

### 2.4. Exercise Decreases Hepatic Triglyceride Content

**Figure 5:**
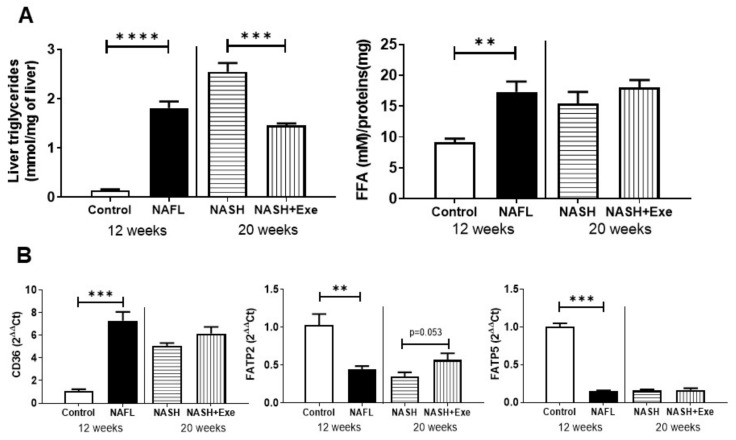

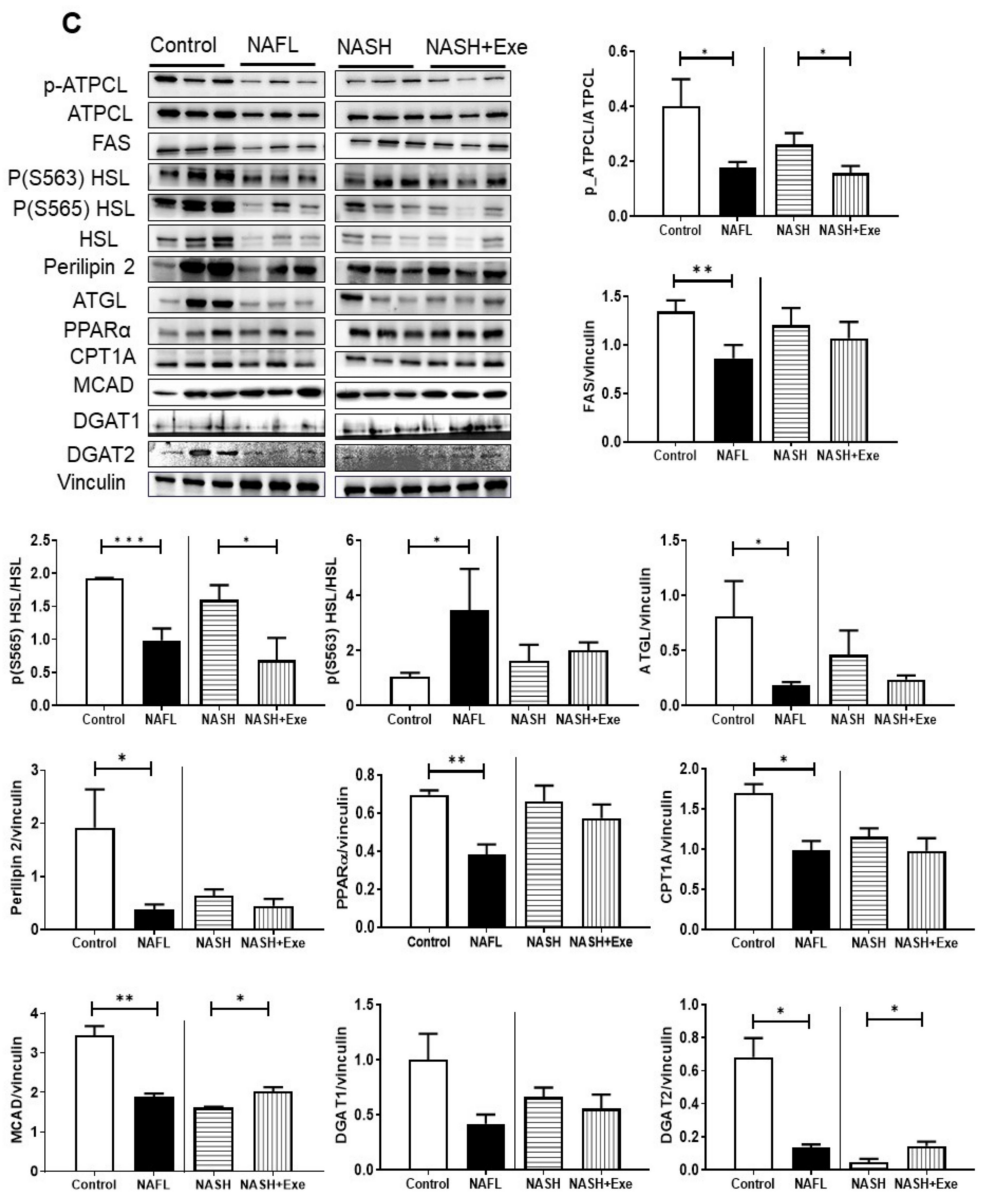
Effect of exercise on hepatic lipid metabolism. (**A**) Liver content of triglycerides and free fatty acids (FFA) were compared in mice fed a control diet or a choline-deficient high-fat diet (CD-HFD) for 12 weeks (NAFL) and in mice fed a CD-HFD for 20 weeks with (NASH + EXE) or without exercise (NASH) *(*unpaired *t*-test; ** *p <* 0.005; *** *p <* 0.001; **** *p <* 0.0001). (**B**) Semi-quantitative PCR measurement of CD36 mRNA and fatty acid transport protein 2 (FATP2) and fatty acid transport protein 5 (FATP5) levels in liver extracts relative to β2-microglobulin. CD36 was higher in NAFL than in controls (*p* < 0.001). FATP5 and FATP2 were lower in NAFL than in controls. (**C)** Immunoblots of liver homogenates comparing control and NAFL mice, and NASH and NASH + EXE groups. The signals from immunoblots were quantified and normalized with vinculin and are reported as mean ± SD (*n* = 3 per group). (ATGL, adipose triglyceride lipase*;* ATPCL, ATP citrate lyase; FAS, fatty acid synthase; HSL, hormone-sensitive lipase; CPT1A, carnitine palmitoyltransferase alpha; MCAD, medium-chain acyl-CoA dehydrogenase (unpaired *t*-test; * *p <* 0.05; ** *p <* 0.005; *** *p <* 0.001). Details of Western blots are given in Figure S2.

The histological evidence of decreased lipid content after exercise was confirmed with biochemical measurements (Figure 5A). After 12 weeks of CD-HFD, hepatic levels of triglycerides rose 13-fold. A further increase at 20 weeks was evident only in sedentary mice, where levels remained significantly higher than those in exercised mice. The hepatic levels of free fatty acids showed a different pattern. After 12 weeks, levels increased but thereafter remained stable and did not change with exercise (Figure 5A). In all mice fed the CD-HFD, the mRNA level of the scavenger receptor and lipid transport facilitator, CD-36, was elevated at least six-fold and was unaffected by exercise (Figure 5B). However, the expression of the long-chain fatty acid carriers fatty acid transport protein (FATP), FATP2, and FATP5 showed the reverse trend (Figure 5C). The mRNA of FATP2 and FATP5 were both decreased by the CD-HFD. Exercise only partially reversed this trend for FATP2.

The hepatic expression of lipogenic enzymes was decreased by the CD-HFD (Figure 5). After 12 weeks, both fatty acid synthase (FAS) and ATP citrate lyase (ATPCL) decreased significantly. Moreover, the phosphorylation of ATPCL was decreased by CD-HFD. Exercise also decreased the ratio of P-ATPCL/ATPCL relative to sedentary livers. Similarly, the hepatic lipid catabolic enzymes were downregulated. CD-HFD severely depressed the levels of hormone-sensitive lipase (HSL), but the balance of phosphorylation at sites 563, relative to 565, was changed by exercise. The expression of adipose triglyceride lipase (ATGL) was generally depressed in all CD-HFD fed livers (Figure 5). The expression of perilipin 2 was decreased and remained depressed by the CD-HFD. The nuclear receptor peroxisome proliferator-activated receptor α (PPARα) and fatty acid catabolism enzymes, CPT1α and MCAD, were generally decreased by the CD-HFD. However, exercise was associated with a modest increase in MCAD relative to sedentary mice. The triglyceride synthesis enzyme DGAT2 was decreased in all CD-HFD livers. 

### 2.5. Exercise Decreases TNFα

**Figure 6:**
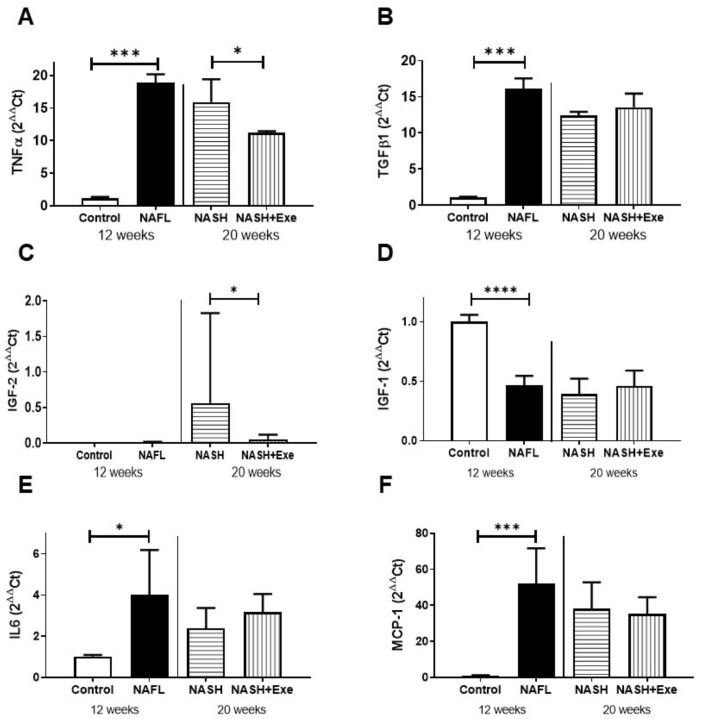
Effect of exercise on expression of liver biomarkers. Semi-quantitative PCR analysis of mRNA expression relative to β2 microglobulin in livers from control, NAFL, NASH, and NASH + EXE groups. (**A**) Tumor necrosis factor α (TNFα) was increased in NAFL vs. control (*p* < 0.001) and decreased in NASH + EXE vs. NASH sedentary (*p* = 0.05). (**B**) Transforming growth factor β (TGFβ1) was increased in the NAFL vs. control group (*p* < 0.001) but not changed in NASH vs. NASH + EXE. (**C**) Insulin-like growth factor 2 (IGF-2) was increased in the NASH sedentary relative to NASH + EXE. (**D**) IGF-1 was decreased by CD-HFD. (**E**) Interleukin-6 (IL6) increased in NAFL. (**F**) Monocyte chemoattractant protein 1 (MCP-1) increased in NAFL. Groups are described in Figure 1 (unpaired *t*-test; *p ≤ 0.05; ***p < 0.001; ****p < 0.0001).

The mRNA expression of several markers of inflammation was measured in the liver. The expression of the proinflammatory cytokine tumor necrosis factor α (TNF α) increased after 12 and 20 weeks of CD-HFD in comparison to controls, but exercise significantly blunted this rise (Figure 6A). The expression of transforming growth factor β1 (TGFβ1) was similarly elevated in all CD-HFD treated livers (Figure 6B). However, exercise did not influence its expression. Insulin-like growth factor 2 (IGF-2) was undetectable in control samples and was significantly elevated in the sedentary NASH group relative to all other groups (Figure 6C). In contrast, IGF-1 was uniformly decreased in all CD-HFD groups and not affected by exercise. Interleukin-6 was increased in the NAFL group relative to controls but not affected by exercise. Similarly, monocyte chemoattractant protein 1 (MCP-1) increased all CD-HFD groups and was not affected by exercise.

### 2.6. CD-HFD Induces ER Stress

**Figure 7:**
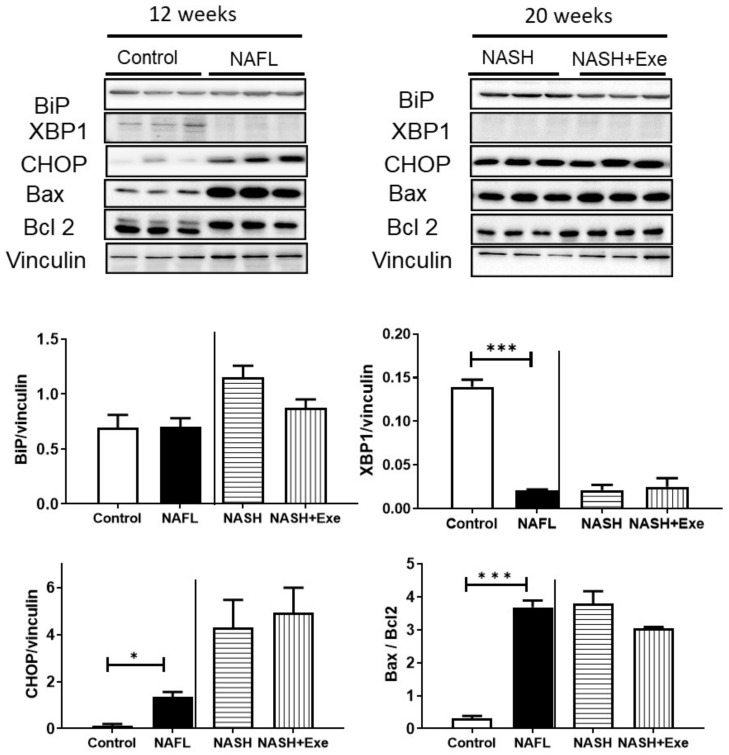
Effect of diet and exercise on endoplasmic reticulum (ER) stress and apoptosis. Immunoblots (upper panel) and quantification (lower panel) of proteins expressed in livers from control, NAFL, NASH, and NASH + EXE groups. Comparisons were made between the control and NAFL groups at 12 weeks, and between the NASH and NASH + EXE groups treated for 20 weeks. Binding immunoglobulin protein (BiP) was not different between groups. X-box binding protein (XBP-1) was lowered in NAFL vs. control and remained very low in both NASH groups. C/EBP homologous protein (CHOP) expression was higher in NAFL than in controls and increased further in the NASH groups. The ratio of Bcl-2 associated X protein (Bax) to Bcl-2 was elevated in NAFL vs. control but was not significantly changed by exercise. Vinculin served as the loading control. Groups are as described in Figure 1 (unpaired *t*-test; *p < 0.05; ***p < 0.001). For more details of Western blots, please view Figure S2.

Selected markers for endoplasmic reticulum (ER) stress were examined by immunoblotting in homogenates of liver. Binding immunoglobulin protein (BiP) tended to decrease in the exercised group (Figure 7). After 12 weeks of CD-HFD, the expression of X-box binding protein 1 (XBP-1s), which results from the inositol-requiring enzyme (Ire)1 mediated splicing of XBP-1 mRNA, was severely downregulated. Levels of XBP-1s remained depressed at 20 weeks in both exercised and sedentary groups. Conversely, after 12 weeks of CD-HFD, the expression of CCAAT-enhancer-binding protein homologous protein (CHOP) was induced. This upregulation was even more pronounced after 20 weeks of CD-HFD and was not affected by exercise. Since CHOP can trigger the intrinsic apoptotic pathway, we quantified the expression of the pro-apoptotic protein, BAX, and the anti-apoptotic protein, BCL2. The CD-HFD was associated with an upregulation of BAX, such that the ratio of BAX to Bcl2 increased significantly (Figure 7). Exercise tended to decrease this ratio.

### 2.7. Exercise Increases Autophagy

**Figure 8:**
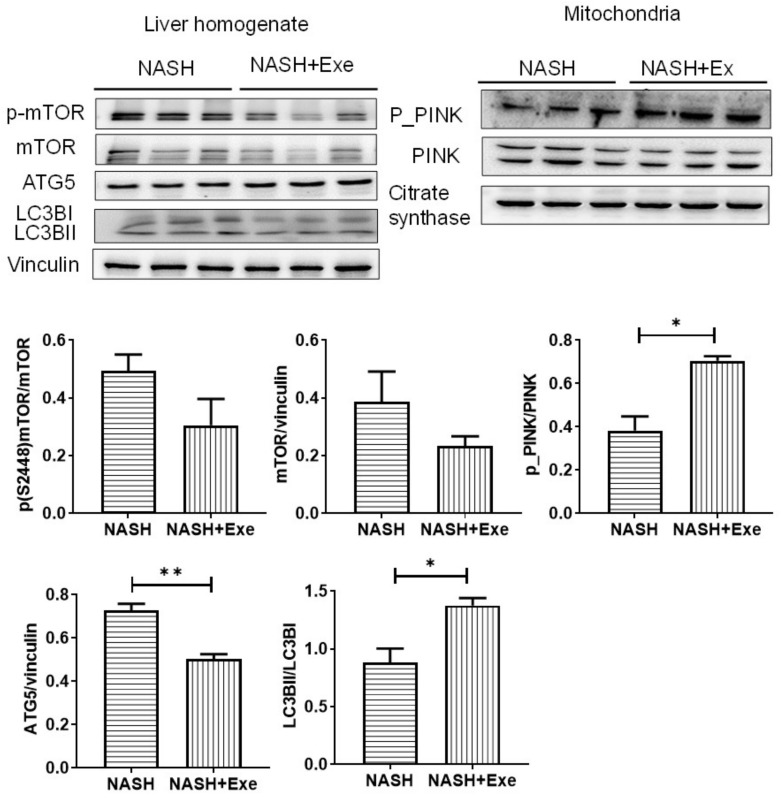
Effect of exercise on autophagy. Immunoblots of protein expression (upper panels) and quantification (lower panels) in liver homogenates (left panel) and mitochondria (right panel) for markers of autophagy in the NASH sedentary and NASH + EXE groups. mTOR and its phosphorylation tended to decrease in the NASH + EXE group. Autophagy related gene 5 (ATG5) tended to decrease in the NASH + EXE group. The ratio of light chain 3BII (LCBII) to LCBI was increased in the NASH + EXE group (*p* < 0.05). Vinculin served as the loading control. The phosphorylation of PTEN-induced kinase (P-PINK) increased in mitochondria of the NASH + EXE group (*p* < 0.05). Citrate synthase served as the loading control. Groups are as described in Figure 1 (unpaired *t*-test; *p < 0.05; **p < 0.005). For more details of Western blots, please view Figure S2.

To evaluate whether the autophagy activation in the liver contributes to the improvement in hepatic steatosis after exercise, we evaluated the level of autophagy-specific microtubule-associated protein light chain 3 (LC3) by immunoblotting of liver homogenate in exercised and sedentary CD-HFD mice. The analysis of LC3BII and LC3BI demonstrated a significantly higher ratio of LC3II/LC3I in the exercised group, which is indicative of an accumulation of autophagosomes (Figure 8). Since mTOR acts upstream to inhibit autophagy, we evaluated the expression of mTOR and its phosphorylation at S2448, which correlates with the activity of mTORC1. Both the expression of mTOR and P-S2448-mTOR were lower in livers from exercised mice than in those of sedentary mice. To evaluate selective autophagy in the mitochondria, we tested for the recruitment of active, phosphorylated PTEN-induced kinase (PINK) to the mitochondrial compartment. P-PINK was higher in the exercise group (Figure 8).

### 2.8. Exercise Decreases Hepatic Nodule Formation.

**Figure 9:**
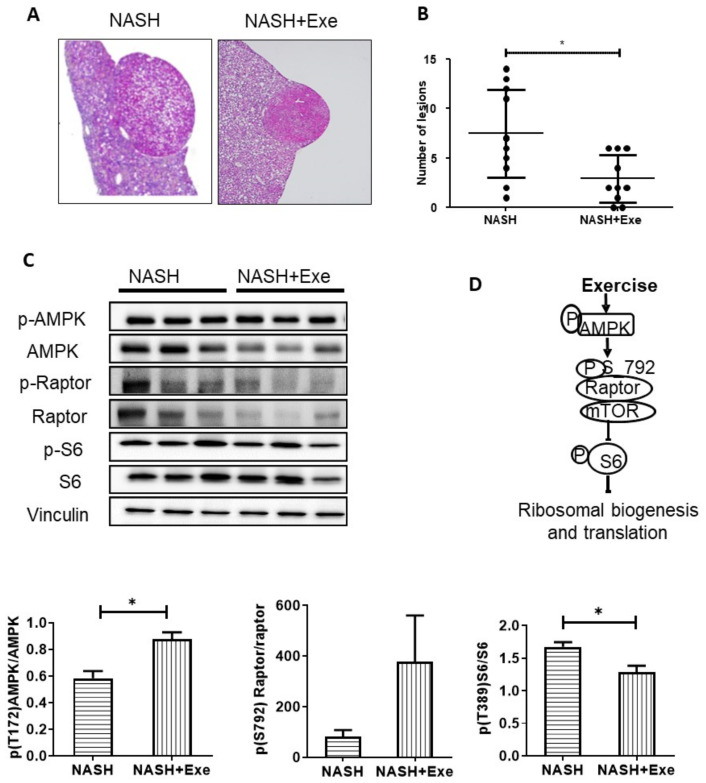
Effect of exercise on liver tumors. (**A**) Representative histological images of hematoxylin and eosin (H&E)-stained liver sections with adenomas in NASH sedentary (left panel) and exercise (right panel) mice (magnification x40). (**B**). Comparison of the number of liver adenomas in NASH sedentary and NASH + EXE mice. NASH + EXE mice developed fewer nodules than did NASH sedentary mice (Mann–Whitney U-test; *p < 0.05). (**C**). Immunoblots showing protein expression and phosphorylation status of AMP-activated protein kinase (AMPK), regulated associated protein of mTOR (Raptor) and ribosomal protein S6. Signals were quantified and normalized for vinculin. Phosphorylation of AMPK was increased and phosphorylation of S6 was decreased in the NASH-EXE group. Groups are as described in Figure 1 (unpaired *t*-test; *p* < 0.05). For more details of Western blots, please view Figure S2. (**D**) Schematic representation of the AMPK–Raptor–S6 cascade.

Extended exposure to a CD-HFD can lead to hepatic tumors. Indeed, all mice in the 20 weeks CD-HFD sedentary group displayed liver nodules, histologically compatible with hepatocellular adenoma, in accordance with the histological tumor classification of the mouse hepatobiliary system [17] (Figure 9A). In particular, the lesions were sharply demarcated from surrounding liver parenchyma, with loss of the normal lobular architecture and irregular, solid, growth patterns. Macrovesicular steatosis was present in most nodules. Despite the presence of hepatocytes of varying sizes, areas of frank cellular atypia, as well as necrosis or blood vessel invasion, were not observed. The incidence of tumor nodules was significantly reduced in the exercised group, wherein only 70% of mice developed liver nodules (SED vs. EXE, *p* < 0.01). Furthermore, exercise negatively affected tumor burden; the mean number of nodules per liver was reduced, 2.9 ± 2.5 vs. 7.7 ± 4.4 for sedentary and exercise, respectively (Figure 9B). To elucidate the mechanism mediating these tumor-suppressive effects of exercise, we examined the AMP-activated protein kinase (AMPK)—mTOR signaling pathway in liver homogenate. First, we quantified the phosphorylation of AMPKα on T172, as a measure of AMPK activation, then the activating phosphorylation of S6, responsible for ribosomal biogenesis and translation (Figure 9C). After 20 weeks of CD-HFD, phosphorylation of AMPK (T172) increased, whereas phosphorylation of S6 decreased in exercised mice (Figure 9C).

### 2.9. Exercise Does not Change Mitochondrial Respiration

**Figure 10:**
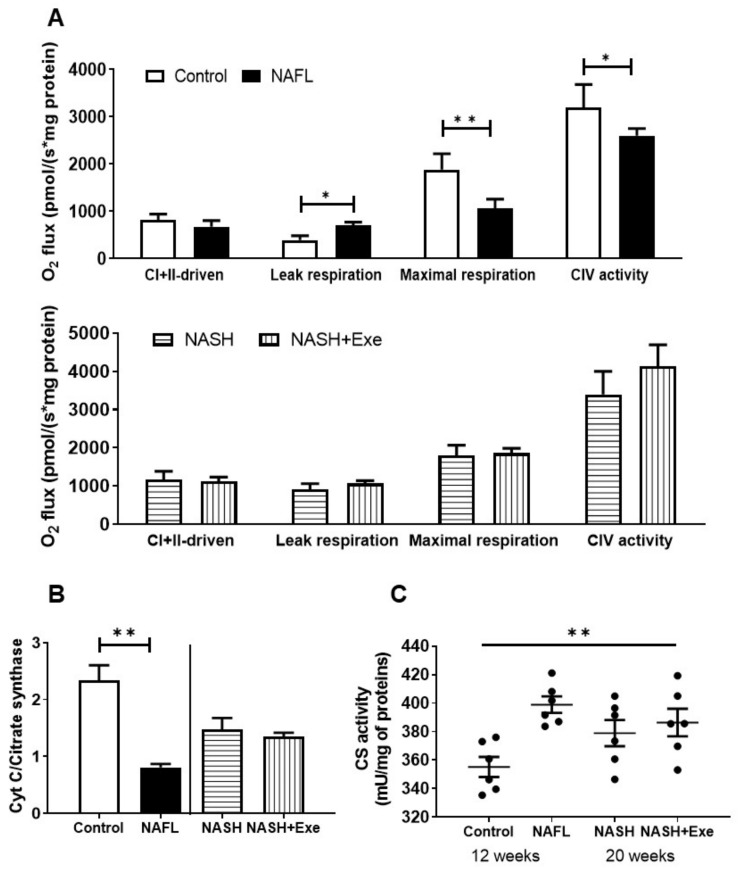

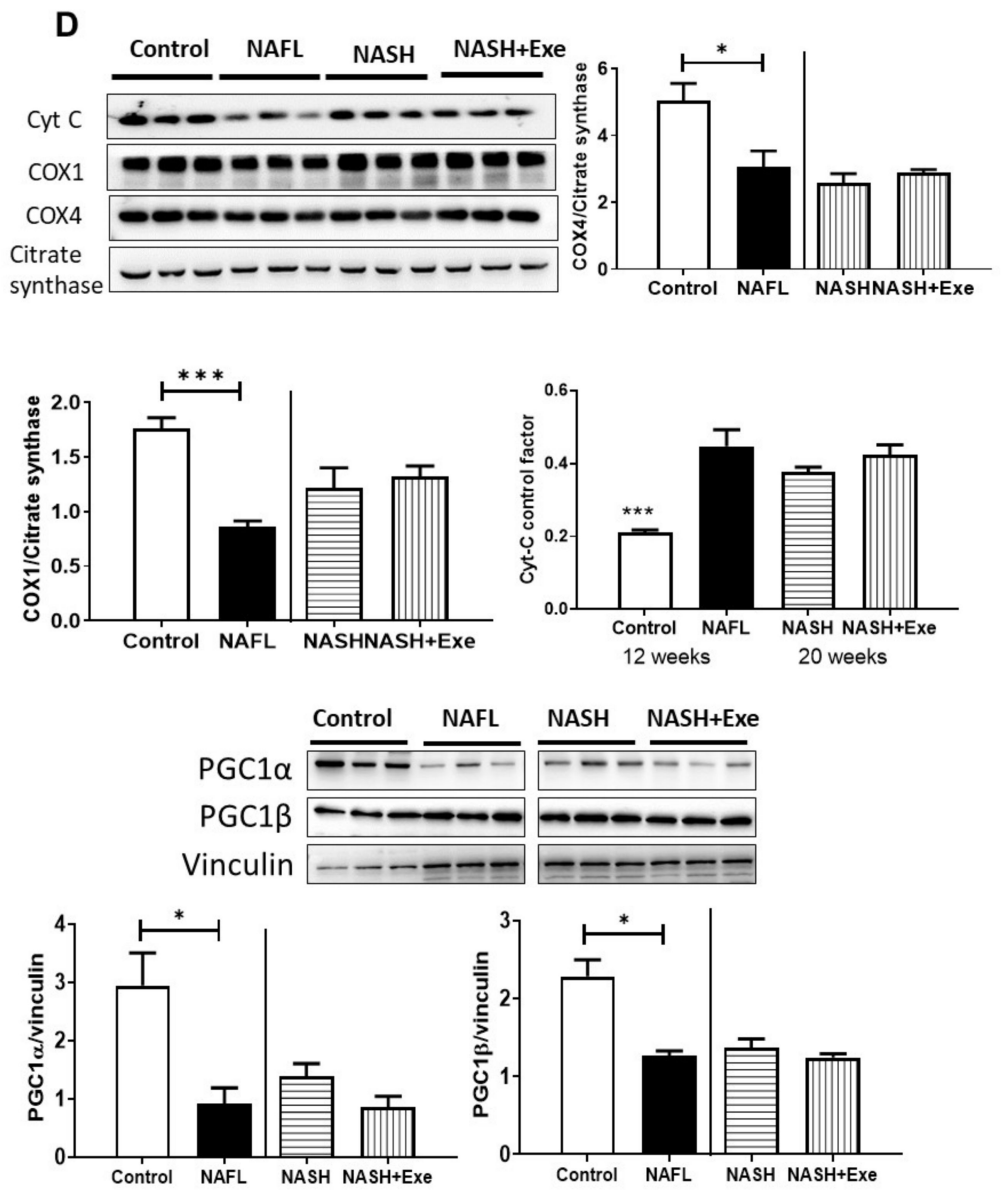
Effect of exercise on mitochondrial bioenergetics. (**A**) High-resolution respirometry of oxygen consumption (O_2_ flux) in mitochondria isolated from livers of control vs. NAFL group (12-week treatment) (left panel) and NASH sedentary vs. NASH + EXE groups (right panel). O_2_ flux was measured with an O2k Oroboros instrument. Mitochondria were exposed to sequential additions of pyruvate/malate, glutamate, succinate, ADP, cytochrome *c*, oligomycin, rotenone, antimycin A, TMPD, ascorbate, and azide. Coupled complex I- and complex II–driven, leak respiration, maximal respiration, and complex IV OCRs were recorded and normalized for protein (*n* = 3 per group). (**B**) Comparison of cytochrome *c* control factors. The ratios were measured as the fractional change of O_2_ flux after the addition of excess cytochrome *c* and calculated as (Flux CI + II_cytc_ -Flux CI + II)/Flux CI + II_cytc_. The ratio was significantly elevated in NAFL and NASH groups relative to control. (**C**) Comparison of mitochondrial citrate synthase activity in control vs. NAFL group and NASH sedentary vs. NASH + EXE groups. Activity was normalized for mitochondrial protein and was higher in NAFL vs. control (one way ANOVA; *p < 0.05). (**D**) Immunoblot showing expression of cytochrome *c* and of cytochrome *c* oxidase (COX) subunits 1 and 4 in mitochondria. Citrate synthase served as the loading control. Cytochrome *c* and COX1 and COX4 were lower in NAFL vs. control groups. Expression of PGC1α and PGC1β was evaluated with vinculin as the loading control (unpaired *t*-test; *p < 0.05; **p < 0.005; ***p < 0.001). For more details of Western blots, please view Figure S2.

Since dysfunctional mitochondria contribute to the progression of NAFLD, we queried whether exercise halted the progression of NAFL to NASH by improving mitochondrial respiration. In respirometry studies, mitochondria from NAFL livers showed no difference in combined complex I- and II-driven respiration but displayed higher leak respiration, an indicator of dissipated membrane potential, and significantly lower maximal respiration and complex IV respiration than did control mitochondria (Figure 10A). These differences were not detected when NASH sedentary and NASH exercise groups were compared. However, the cytochrome *c* control factor was significantly increased relative to control in all NAFL and NASH groups (Figure 10B). In addition, citrate synthase (CS) enzyme activity and expression were monitored as an indicator of the tricarboxylic acid cycle (Figure 10C,D). Whereas the protein expression of CS remained constant in all groups, the activity increased in NAFL relative to control (Figure 10C) but not in exercise relative to sedentary. To determine whether a decrease in cytochrome *c* content could account for the increase in cytochrome *c* control factor, we quantified the protein expression in mitochondria. The expression of cytochrome *c* decreased in NAFL relative to control but remained constant in NASH sedentary relative to exercise (Figure 10D). To test whether the decrease in maximal respiration and complex IV respiration could be attributed to the downregulation of respiratory proteins, we quantified the expression of cytochrome c oxidase (COX) subunits 1 and 4. The expression of both COX subunits was lower in NAFL than in controls but similar in a comparison between NASH sedentary and exercise groups (Figure 10D). To ascertain whether mitochondrial biogenesis was modified in the liver, we measured levels of PGC1α and PGC1β that were decreased slightly compared to controls (Figure 10D).

### 2.10. The CD-HFD Promotes Sinusoidal Endothelial Cell Defenestration

**Figure 11:**
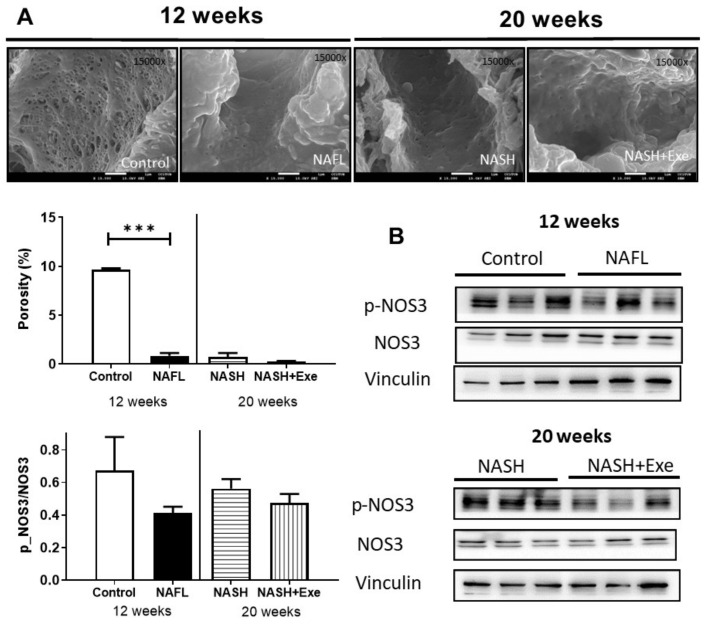
Effect of exercise on liver endothelium. (**A**) Scanning electron microscopy images of sinusoids showing endothelial cells from livers of control, 12-week CD-HFD treated (NAFL), 20-week CD-HFD sedentary (NASH), and 20-week CD-HFD exercised mice (magnification ×15,000) (upper panel). Porosity, defined as the percentage of endothelial cell membrane perforated by fenestrations, was quantified (bottom panel) (unpaired *t*-test; ***p < 0.001). (**B)** Immunoblots of the gene product of nitric oxide synthase 3 (*NOS3*) and its phosphorylated form expressed in liver homogenates of control and NAFL groups (12 weeks treatment), and NASH and NASH + EXE groups (20-week treatment). Immunoblots were quantified and normalized with vinculin. The NAFL, NASH, and NASH + EXE groups are described in Figure 1. For more details of Western blots, please view Figure S2.

After 12 weeks of CD-HFD, the fenestration of the liver endothelium was reduced 10-fold, as determined by the decrease in porosity of the sinusoidal endothelial cells (Figure 11). This morphological change featured throughout the 20 weeks of CD-HFD and was not affected by exercise. The expression of the gene product of *NOS3,* endothelial nitric oxide synthase (eNOS), did not change, but its phosphorylation tended to decrease in all CD-HFD treated livers, although this did not reach statistical significance.

### 2.11. Response of Skeletal Muscle to Exercise under CD-HFD

**Figure 12:**
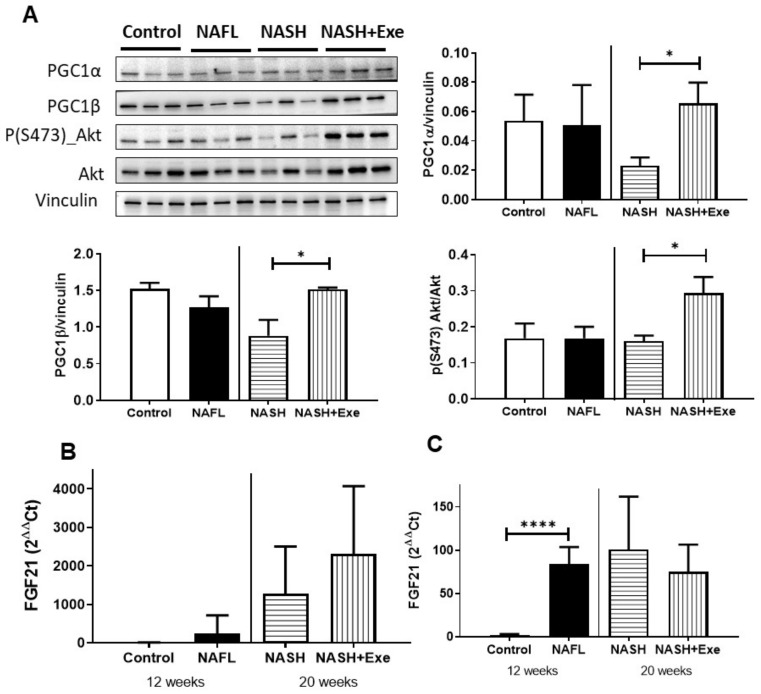
Effect of exercise on skeletal muscle. (**A**) Immunoblots of peroxisome proliferator-activated receptor gamma coactivator 1α and 1β- (PGC1α, PGC1 β) and phosphorylated Akt in skeletal muscle. (**B**) FGF21 mRNA expression in skeletal muscle. (**C**) FGF21 mRNA expression in liver. Vinculin served as the loading control (unpaired *t*-test; *p < 0.05; ****p < 0.0001). For more details of Western blots, please view Figure S2.

Exercise is well known to induce mitochondrial biogenesis in skeletal muscle. To test whether mice treated with the choline-deficient HFD respond to exercise as expected, we compared the expression of transcription coactivator proteins, PGC1α and PGC1β in skeletal muscle in control, and CD-HFD treated mice (Figure 12A). Both PGC1α and PGC1β were increased by exercise relative to the sedentary group, suggesting an increase in mitochondrial content. The exercise was also associated with the activation of the AKT signaling pathway in skeletal muscle (Figure 12A). Since skeletal muscle secretes peptides affecting liver function, we measured the mRNA expression of fibroblast growth factor 21 (FGF21), which can be released by both skeletal muscle (Figure 12B) and liver (Figure 12C). In skeletal muscle (Figure 12B), the CD-HFD increased FGF21 with exercise, further augmenting expression. Conversely, in the liver, the increase relative to controls was less pronounced, and the expression of FGF21 tended to decrease with exercise.

## 3. Discussion

In the present study, we explored the selective benefits of physical activity as a means of modifying the outcome of NAFLD triggered by a high-fat diet. We report that daily exercise alone attenuates the transition from NAFL to NASH, reduces the hepatic accumulation of triglycerides, impedes the progression of fibrosis, and decreases the incidence of tumor formation. 

Previous studies evaluating the effect of lifestyle interventions have relied on post-intervention liver biopsy to gauge the improvement in histologic features of NASH [7,18,19,20]. However, because dietary changes designed for weight loss and exercise programs were inextricably linked, improvement in liver function attributable to exercise could not be discerned. Conversely, our study design has circumvented this ambiguity, and we report that exercise without dietary intervention lessens ballooning, a hallmark of NASH, and arrests the fibrotic progression of disease leading to HCC. Since these beneficial effects of an imposed exercise regimen occurred without concomitant weight loss (Figure S1), we claim that exercise is a positive therapeutic measure despite the continued burden of overnutrition. 

The experimental model we selected was a non-obesogenic, nutrient-deficient, high-fat diet model (Table S1), which offers the advantages of a rapid onset of disease and mirrors the complexity of clinical NASH with fibrosis and development of tumors [15,16,21] but obviates the influence of weight gain [15] and peripheral insulin resistance [22,23]. Hence, it was a robust choice in which to test the short-term effects of regular exercise. In addition, choline-deficiency presents the added liability of a disturbance in the phosphatidylcholine content of hepatic membranes, including mitochondrial membranes, which is a source of mitochondrial dysfunction [24] and contributes to the pathogenesis of NAFLD in this experimental model.

Exercise lowered hepatic levels of triglycerides in our CD-HFD mice (Figure 2D and Figure 5A), which is in line with non-invasive data collected from clinical studies [25]. The genesis of steatosis in NAFLD is multi-factorial and could arise from increased fatty acid uptake, increased lipogenesis de novo [26], decreased β-oxidation, impaired triglyceride lipolysis or defective assembly and secretion of VLDL particles. In our CD-HFD mice, an increase in fatty acid uptake by CD36 at the expense of FATP2 and FATP5 was presumed, given the upregulation of CD36 gene expression and the downregulation of FATP2 and FATP5. The upregulation of CD36 is consistent with the increase in plasma and hepatic FFA levels (Figure 4 and Figure 5) and is a feature observed in other experimental models [25,26]. This increased CD36 expression promotes the accumulation of unsaturated FAs, which are a driving force for steatotic triglyceride formation [27].

Unlike CD36, the transcriptional downregulation of both FATP2 and FATP5 was not expected. In a study of mice fed a choline-sufficient HFD for 12 weeks, FATP2 and 5 were unchanged [28]. However, when mice were fed a lipogenic methionine choline-deficient diet for 4 weeks, FATP2 and 5 were marginally decreased, although increases in FATP 1 and FATP4 were noted [29]. Apart from any dietary stimulus, the genetic deletion of FATP2 in the liver provokes commensurate increases in CD36 and FATP1 expression, indicating compensatory mechanisms to coordinate and regulate the uptake of lipids [27]. Although we did not investigate the complete profile of fatty acid uptake systems in our CD-HFD model, we deduce that the negative correlation between exercise and hepatic lipid accumulation was independent of the changes in FATP2, FATP5, and CD36. Increased lipogenesis de novo was not a feature of our CD-HFD experimental model [26], since lipogenic enzymes were downregulated (Figure 5). In fact, exercise extended this downregulation further by suppressing the phosphorylation of ATPCL. Impaired lipolysis of hepatocyte triglyceride stores likely contributed to steatosis in our model, since two lipases involved in the sequential hydrolysis of triglycerides, ATGL and HSL, were downregulated in all groups of CD-HFD (Figure 5). Exercise did not change this trend. As a counter-measure to cytoplasmic lipid storage within droplets, expression of perilipin 2 tended to decrease in all groups of CD-HFD (Figure 5), thus rendering triglycerides more available to catabolism via autophagy [30]. Hence, since autophagy is, in turn, stimulated by exercise [31] (Figure 8), we attribute the lower steatosis in the CD-HFD exercised group in part to an increase in autophagic processes. In addition, the modest reduction in PPARα combined with those of CPT1α and MCAD protein levels in the NAFL group suggest that a decrease in fatty acid beta-oxidation could contribute to steatosis in this CD-HFD model. Although slight, exercise was associated with an upregulation of MCAD protein (Figure 5). The downregulation of DGAT2 was more pronounced (Figure 5). While the upregulation of DGAT2 has been associated with increased triglyceride synthesis [32], the consequences of its downregulation in the liver, as observed here, is unclear [33]. DGAT2 has been ascribed a role in VLDL-triglyceride secretion. Therefore, we cannot exclude that exercise has partly reversed the CD-HFD induced DGAT2 suppression and improved VLDL secretion from the liver.

In NAFLD, proinflammatory TNFα released by Kupffer cells and steatotic hepatocytes mediates liver injury in part by activating NFkB signaling pathways in stellate cells [27,30]. Exercise reduced the TNFα release elicited by CD-HFD (Figure 6A). Reduced levels of TNFα have been linked to an amelioration of NAFLD, since anti-TNFα antibodies were reported to improve liver histology, to reduce circulating levels of AST and ALT, and to diminish hepatic fat content [34]. Moreover, a correlation has been shown between plasma levels of TNFα and the presence of ballooned hepatocytes [35]. Thus, we find that notwithstanding the continued CD-HFD diet, exercise prompted a decline in hepatocyte steatosis combined with an attendant decrease in TNFα expression leading to a drop in the incidence of ballooning (Figure 2B) and, consequently, to a lower NAFLD activity score (Figure 2C) along with decreases in ALT and AST concentrations (Figure 4). A novel biomarker, IGF-2, is purported to be negatively correlated with the extent of NAFLD and, in particular, to the degree of ballooning [35]. However, in our model, IGF-2 was undetectable in control livers, was highest in the sedentary 20-week CD-HFD group linked to the highest NAFLD score, and was significantly reduced in the exercised group (Figure 6C). Consequently, IGF-2 was positively rather than negatively correlated with disease. In fact, IGF-2 was reported to increase in tumor-bearing livers of mice subjected to a choline-deficient diet and CCl_4_ treatment [36]. For this reason, the reduced levels of IGF-2 produced in the exercise group likely reflect the fewer numbers of nodules formed. Unlike IGF-2, the levels of IGF-1 were negatively correlated with NASH (Figure 6D). This observation is consistent with clinical reports of NAFLD [37] and with experimental findings in choline-deficient diets [38]. Similarly, neither liver-derived interleukin-6 nor MCP-1 could distinguish exercise from sedentary NASH livers.

The ability of exercise to prevent the histological progression of fibrosis (Figure 3A) was confirmed by the changes in circulating markers of hepatic fibrosis, namely, Pro-C3, Pro-C4, and C6M (Figure 3C). Certainly, the histological assessment of fibrosis correlated well with these circulating markers [39]. Pro-C3, a defined epitope of the NH2-terminal propeptide of type III procollagen, is a marker of active fibrogenesis and is released by the protease ADAMTS-2 during collagen maturation, which is a prerequisite for efficient incorporation of collagen type III into collagen fibrils [40]. Pro-C4, a marker of collagen type IV formation, reflects pericellular fibrosis and not bridging reticular fibrotic bands, as does Pro-C3 [41]. C6M detects an internal epitope in the collagen type VI that is exposed by multiple matrix metalloproteinases when the collagen structure is degraded [42]. It is severely upregulated in the fibrotic space of Disse and portal tract stroma and engages in signaling related to the metabolic syndrome and fibrogenesis [42]. These fibrosis biomarkers were all significantly decreased in exercised mice compared to sedentary mice (Figure 3C). In fact, the plasma concentrations of all three biomarkers in the 20-week CD-HFD exercised group were comparable to those of the 12-week NAFL group.

Given that fibrosis plays a role in the pathogenesis of HCC and its presence portends a poor prognosis, the significantly lower extent of fibrosis in the exercised CD-HFD groups (Figure 4) predicts that the development of NASH-related tumors would be attenuated in this group. As expected, both the number of animals bearing tumors and the number of hepatocellular adenomas per liver were significantly reduced in exercised mice (Figure 9). In an earlier long term study of a similar CD-HFD diet administered to C57Bl/6J mice, Yoshida et al. queried whether features of the disease spectrum of NAFL-NASH-HCC could be reversed if a standard diet was imposed for 12 weeks after a 36-week regimen of a CD-HFD. Although steatosis and lobular inflammation regressed with a change in diet and fibrosis was partially reversible, the incidences of hepatocellular adenoma and carcinoma progressed [16]. This reveals a difference between the strategies of enforcing an exercise regimen while a deleterious diet continues to expose the liver to the acute consequences of high fat and of merely restricting the offending diet once the inexorable alterations in the molecular machinery, leading to HCC, have taken root. We have previously shown exercise to carry benefits in other models of HCC. For instance, regular physical activity significantly decreases the occurrence of tumors, from 100% down to 70%, in a model of NASH induced by the ablation of hepatocellular PTEN [13]. We also confirmed that the exercise-related mechanism was the activation of AMPK and inhibition of the mTOR/S6K pathway [13,14,37]. This mechanism holds for the current model of dietary-induced NASH. Exercise increased the activating phosphorylation of AMPK, resulting downstream in less phosphorylation of S6 (Figure 9D). However, additional mechanistic changes in other pathways cannot be excluded. 

The autophagy machinery is relevant when considering tumor development and progression. Autophagy can be modified in both genetic and dietary models of obesity. It is suppressed in the liver, at least in part due to a reduction in the expression level of key autophagy molecules, such as Atg7 [38,39]. In fact, mice with systemic mosaic deletion of Atg5 and liver-specific deletion of Atg7 develop multiple liver tumors [31]. Autophagy deficiency is accompanied by defective insulin signaling and elevated ER stress. In our dietary model, the ER stress was chronic, as reflected by the absence of XBP1 expression and the induction of the pro-apoptotic transcription factor CHOP that transactivates pro-apoptotic proteins (Figure 7). As expected, evidence of an activated pro-apoptotic pathway was detected since Bax was upregulated, and the ratio of Bax to Bcl2 was significantly elevated. However, exercise relieved the autophagic block that can accompany ER stress. Autophagy appeared to be activated by exercise, as shown by the tendency for mTOR downregulation, an increase in LC3BII/LC3BI, and an increase in mitochondrial recruitment of phosphorylated PINK indicative of mitophagy (Figure 8). The benefit of an activated mitophagy process derives from the need to clear hepatocytes of dysfunctional mitochondria that accumulate in NASH [41,42]. Indeed, mitochondria in our CD-HFD model were compromised (Figure 10). The cytochrome *c* control factor was significantly increased in all respirometry runs, likely because of activation of the pro-apoptotic pathway, loss of cytochrome c, and mitochondrial uncoupling (Figure 10). In addition, the decrease in maximal respiration and complex IV activity is perhaps explained by modest changes in the expression of at least two components of complex IV. Overall, our findings support the general view that exercise stimulates selective autophagic processes in the liver to alleviate hepatocytes of its deleterious burden of lipid overload and dysfunctional mitochondria [43].

LSECs undergo morphological and functional changes in NAFLD, leading to sinusoidal capillarization [44]. These phenotypic changes stemming from endothelial defenestration appear early, are features of most experimental models of NASH [45], and have been linked to exposure to dietary fat and circulating free fatty acids [46]. With the progression of NAFLD, LSECs acquire a proinflammatory phenotype and thereby become effectors of liver inflammation in NASH and promoters of fibrosis [47]. LSECs have also been attributed a pro-oncogenic role in HCC through their release of the adipokine fatty acid binding protein-4, which in turn induces hepatocyte proliferation [48]. In our design, sinusoidal capillarization was firmly established at the 12-week NAFL stage before exercise was implemented, and exercise did not reverse the morphological changes in LSECs (Figure 11). Therefore, the anti-tumorigenic actions of exercise are unlikely to implicate pathways central to LSECs.

Exercise directly affects skeletal muscle, as shown by the upregulation of PGC1α and PGC1β and activation of Akt and avenues exist for crosstalk between muscle and liver. We have probed FGF21 as a candidate secreted by both tissues and which is exercise responsive. FGF21 is beneficial in NASH [49], and muscle-derived FGF21 could wield hepatic effects, given that in our CD-HFD model the expression in the muscle, but not the liver, increased in response to exercise (Figure 12). Regardless of the mechanism, our findings show that exercise can change the outcome of NAFLD. Exercise offers both protective and therapeutic effects as it intervenes to decrease triglyceride accumulation in hepatocytes even though nutritional overload persists. Consequently, the decrease in proinflammatory cytokines, hepatocyte ballooning, and fibrosis curb the incidence of tumor formation. In parallel, exercise mitigates tumor progression through a decrease in phosphorylated ribosomal protein S6.

## 4. Methods

### 4.1. Ethics Statement

Mice received humane care and experiments were approved by and conducted according to the regulations of the Bern Animal Welfare Committee, Canton of Bern, Switzerland (BE132/17: 29699 dated 19 February 2018).

### 4.2. Study Design and Animals

Male C57Bl/6N mice (Charles River, Freiburg, Germany) were chosen to avoid the *Nnt* (nucleotide nicotinamide transhydrogenase) mutation carried by C57BL/6J mice. Loss of NNT enzymatic activity has been linked to reduced mitochondrial NADPH/NADP+ ratio, mitochondrial redox abnormalities [50], as well as impaired mitochondrial peroxide metabolism [50] and glucose homeostasis [51]. Mice aged 8 weeks were housed under controlled temperature (22 ± 2 °C) and lighting (12-h light–dark cycles), acclimatized to the facility for one week, then randomly assigned to one of the following four groups and subjected to a diet and activity protocol (Figure 1): (1) mice (*n* = 11) fed a standard diet for 12 weeks (control group); (2) mice (*n* = 11) fed a choline-deficient high-fat diet (CD-HFD) for 12 weeks (baseline NAFL group); (3) mice (*n* = 11) fed CD-HFD for 20 weeks but without exercise (CD-HFD sedentary group); mice (*n* = 11) fed CD-HFD for 20 weeks but with treadmill exercise from weeks 13 to 20 (CD-HFD exercise group) (Figure 1). Food intake and body weight were monitored weekly.

### 4.3. Dietary Intervention

All mice were fed ad libitum. The CD-HFD contained 9% protein, 60% fat, including 2% cholesterol and 31% carbohydrate (HF-CDAA diet, E15673-94, Ssniff Spezialdiäten GmbH, Germany) (Table S1). The standard chow diet contained 12% protein, 16% fat, and 72% carbohydrate (Control diet, E15668-04, Ssniff Spezialdiäten GmbH).

### 4.4. Exercise Protocol

After 12 weeks, the CD-HFD exercise mice were placed on a treadmill (running speed of 12.5 m/min) (Förderband GFB, Elmotec, Kleindöttingen, Switzerland) for 60 min from 08.00 h to 09.00 h, corresponding to their waking time. The exercise was imposed 5 days/week for 8 weeks. Sedentary mice remained in their cages.

### 4.5. Animal Euthanasia

The control and baseline NAFL groups were killed after 12 weeks. The CD-HFD sedentary and exercise groups were killed after 20 weeks, 2 days after the last exercise session. Mice were weighed (Figure S1), then anesthetized deeply with pentobarbital (100 mg/kg i.p.), and blood was collected from the inferior vena cava into heparinized tubes then centrifuged (3000× *g*, 15 min, 4 °C). Plasma was stored at −80 °C for less than 1 month. Before anesthesia, tail blood lactate and glucose levels were measured with a Lactate Scout Analyzer (Senslab, Leipzig, Germany) and an automated glycemia reader (Ascensia Contour, Bayer Health Care, Zürich, Switzerland). After euthanasia, liver tissue was weighed and divided and either immediately snap-frozen in liquid nitrogen or placed in RNA*later* (Sigma-Aldrich R0901, St. Louis, MO, USA) and stored at −80°C, or fixed in 4% phosphate-buffered formaldehyde. Hepatic tumors were counted, sized, and fixed. 

### 4.6. Plasma Analyses

Activity of alanine transaminase (ALT) and aspartate transaminase (AST), and concentrations of triglycerides, total cholesterol, and bile acids were measured (Cobas analyzer 8000, Roche Diagnostics GmbH, Mannheim, Germany). PRO-C3, the N-protease mediated cleavage of the N-terminal propeptide of type III collagen, PRO-C4, an internal epitope in the 7S domain of type IV collagen, and C6M, a neo-epitope of the proteolytic degradation of type VI collagen, were measured by means of a competitive enzyme-linked immunosorbent assay (Nordic Bioscience, Herlev, Denmark), as described [52].

### 4.7. Histology

Formaldehyde-fixed, paraffin-embedded liver tissues were stained with hematoxylin and eosin (H&E) and examined for steatosis, NASH lesions, and tumors by a pathologist blinded to treatment conditions (*LMT*). The NAS score was determined as previously defined by Kleiner et al. [53]. The degree of fibrosis was assessed on sections stained with Sirius Red and visualized with a panorama scanner and case viewer (3D Histech) and 10× objective. Eight digital images were collected from different areas of the left, median, and right lobes, and the signals were quantified with MetaMorph® image analysis software (Molecular Devices, Sunnyvale, CA, USA). Tumor types were assessed as previously described [17]. Oil Red O staining was performed on frozen sections as previously described [54].

### 4.8. Hepatic Triglycerides and Free Fatty Acids

Total triglyceride content was measured with the PicoProbeTM triglyceride fluorometric assay (BioVision, Milpitas, CA, USA). Total free fatty acid content was quantified by means of the fluorometric FFA kit (BioVision).

### 4.9. Tissue Lysis and Immunoblot Analysis

Livers were homogenized in RIPA buffer (150 mM NaCl, 1% NP-40, 0.5% Na-deoxycholate, 0.1% SDS, and 50 mM Tris-HCl pH 7.4) containing protease and phosphatase inhibitors (Roche, Rotkreuz, Switzerland). Protein concentration was measured with the Pierce^TM^ BCA assay (Thermo Fisher Scientific, Rockford, IL, USA). Equal amounts of proteins were separated by sodium dodecyl sulfate polyacrylamide gel electrophoresis (SDS-PAGE) and transferred to nitrocellulose membranes, blocked for 1 h with 5% nonfat milk or BSA, then incubated overnight at 4 °C with primary antibodies (Table S2). After incubation with peroxidase-conjugated secondary antibody (Thermo Fisher Scientific, Rockford, IL, USA), signals were revealed with enhanced chemiluminescence (Amersham ECL Prime, GE Healthcare, Glattburg, Switzerland) and a Fusion CCD camera coupled to a computer equipped with Fusion Capt Fx Software (Vilber-Lourmat, Marne-la-Vallée, France). Signals were quantified with the Bio-1D Advanced software (Vilber-Lourmat). 

### 4.10. Isolation of Total RNA and Quantitative PCR

Total RNA was extracted with the RNeasy Mini Kit (Qiagen, Hombrechtikon, Switzerland) and stored at −80 °C. RNA was reverse-transcribed (SuperScript III Reverse Transcriptase, Invitrogen, Basel, Switzerland). The gene primer, FAM-labelled probe and the TaqMan Universal PCR Master Mix were obtained from Applied Biosystems (Beverly, MA, USA) (Table S3). Amplification was performed with a CFX Connect Real-Time System (Bio-Rad, Hercules, CA, USA). The ΔCt values were calculated relative to β2-microglobulin as the housekeeping gene. Values are triplicates and are reported as fold increase or decrease relative to the controls and calculated as 2^-ΔΔCt^.

### 4.11. Scanning Electron Microscopy (SEM)

Livers were perfused through the portal vein with fixation solution (2.5% glutaraldehyde, 2% formaldehyde, 2 mM CaCl2, 2% sucrose and 0.1 M sodium cacodylate (pH 7.4)) for 5 min. Fixed tissue was cut in blocks (1 × 1 × 5 mm) and stored in 2% formaldehyde at 4 ℃ until processed. For SEM, fixed livers were treated with 1% osmium tetroxide, dehydrated in a graded series of ethanol, and dried. The sections were coated with platinum/palladium and visualized under an S-4700 electron microscope (Hitachi, Tokyo, Japan). For evaluation of capillarization, the percent of open space area in the liver sinusoidal endothelial cells (LSECs; porosity) was measured in 15 randomly selected fields at ×10,000 magnification on at least three animals per group, using ImageJ software.

### 4.12. Respiration in Isolated Liver Mitochondria

Oxygen flux was measured in freshly isolated mitochondria by respirometry (Oxygraph-2k; Oroboros Instruments, Innsbruck, Austria). Mitochondria (200 µg) were added to 2 mL of respiration buffer (110 mM sucrose, 60 mM K+-lactobionate, 0.5 mM EGTA, 3 mM MgCl_2_, 20 mM taurine, 10 mM KH_2_PO_4_, 20 mM HEPES (pH 7.1), at 37 °C). Oxidative phosphorylation was estimated with complex I (pyruvate 5 mM, malate 2 mM, glutamate 5 mM) and complex II (succinate 10 mM) substrates in the presence of ADP (2.5 mM). Leak respiration was recorded after the addition of oligomycin (2.5 μM). For maximum uncoupled respiration, the protonophore carbonyl cyanide m-chlorophenyl hydrazine (CCCP) was titrated in 0.5 μM increments until maximal stimulation of respiration. The protocol was terminated by assessing non-mitochondrial respiration with the complex I and III inhibitor, rotenone (0.5 mM), and antimycin A (2.5 mM), respectively. Finally, the activity at complex IV was recorded with the artificial substrate N,N,N9,N9-tetramethyl-p-phenylenediamine dihydrochloride (TMPD; 0.5 mM) and ascorbic acid (2 mM), and inhibited with azide (100 mM). The cytochrome *c* control factor was measured after simulation of respiration with exogenous cytochrome *c* (10 µM). Respiration states were corrected for non-mitochondrial respiration, and complex IV activity was corrected for azide inhibition. Values were normalized for protein, as described previously [54].

### 4.13. Statistical Analysis

Data are presented as the mean values ± standard deviations (SD). Statistical comparisons were made between control and CD-HFD groups terminated at 12 weeks, and between SED and EXE groups terminated at 20 weeks, except for assessment of cytochrome *c* control factor and citrate synthase activity, where all four groups were compared. The normality of data was assessed by the Kolmogorov–Smirnov test. The nonparametric Mann–Whitney U-test was applied in the case of non-normal distributions. Fischer’s exact test was applied to frequency tables. A *p-*value ≤0.05 was considered statistically significant.

## 5. Conclusions

Our work complements the small number of studies that have evaluated the positive effect of lifestyle interventions on the histological features of NASH [18,19,20]. In addition, our study confirms in yet another model that exercise not only arrests the development of liver tumors [13] but attenuates progression [14]. Furthermore, we have reinforced the notion that the benefit of exercise in suppressing tumors is sustained in NAFLD well after sinusoidal capillarization, ER stress, apoptotic processes, and evidence of mitochondrial dysfunction are established. Finally, we provide direct evidence that exercise alone can be a therapeutic measure and not only a preventive measure in NAFLD, and this should offer hope to patients who fail at sustained, consequential dietary changes, a situation routinely confronting the clinician [55].

## Supplementary Materials

The following are available online at https://www.mdpi.com/2072-6694/12/6/1407/s1, Figure S1: Comparison of body weight and of liver to body weight ratios; Figure S2: Unabridged western blot images; Table S1: Composition of standard and choline-deficient high-fat diets; Table S2: Description and source of antibodies used in the study; Table S3: Description and source of semi-quantitative PCR reagents.

## Abbreviations

ALT	alanine transaminase
AMPK	AMP-activated protein kinase
AST	aspartate transaminase
ATGL	adipose triglycerides lipase
ATPCL	ATP citrate lyase
Bax	Bcl-2 associated X protein
BiP	Binding immunoglobulin protein
CCCP	carbonyl cyanide m-chlorophenyl hydrazine
CD-HFD	choline-deficient high-fat diet
CHOP	CCAAT-enhancer-binding protein homologous protein
CS	citrate synthase
eNOS	endothelial nitric oxide synthase
ER	endoplasmic reticulum
EXE	exercise
HCC	hepatocellular carcinoma
FAS	fatty acid synthase
FATP	fatty acid transport protein
FFAs	free fatty acids
H&E	hematoxylin and eosin
HSL	hormone-sensitive lipase
IGF2	insulin-like growth factor 2
Ire	inositol-requiring enzyme
LC3	light chain 3
LSECs	liver sinusoidal endothelial cells
NAFL	non-alcoholic fatty liver
NAFLD	non-alcoholic fatty liver disease
NAS	NAFLD activity score
NASH	non-alcoholic steatohepatitis
PINK	PTEN-induced kinase
SD	standard deviations
SED	sedentariness
SEM	scanning electron microscopy
TGF	transforming growth factor
TMPD	N,N,N9,N9-tetramethyl-pphenylenediamine dihydrochloride
TNF	tumor necrosis factor
XBP-1	X-box binding protein 1
