# Supplementary material for: Exercise Attenuates the Transition from Fatty Liver to Steatohepatitis and Reduces Tumor Formation in Mice
Source: Cancers (Basel). 2020 May 29;12(6):1407. doi: 10.3390/cancers12061407 (PMC7352494; doi:10.3390/cancers12061407)
Supplement: Supplementary file 1 [file cancers-12-01407-s001.pdf]

Supplementary Materials

Exercise attenuates the transition from fatty liver to steatohepatitis and reduces tumor formation in mice

Maria Guarino, Pavitra Kumar, Andrea Felser, Luigi M. Terracciano, Sergi Guixé-Muntet, Bostjan Humar, Michelangelo Foti, Jean-Marc Nuoffer, Marie V. St-Pierre and Jean-François Dufour

Figure S1

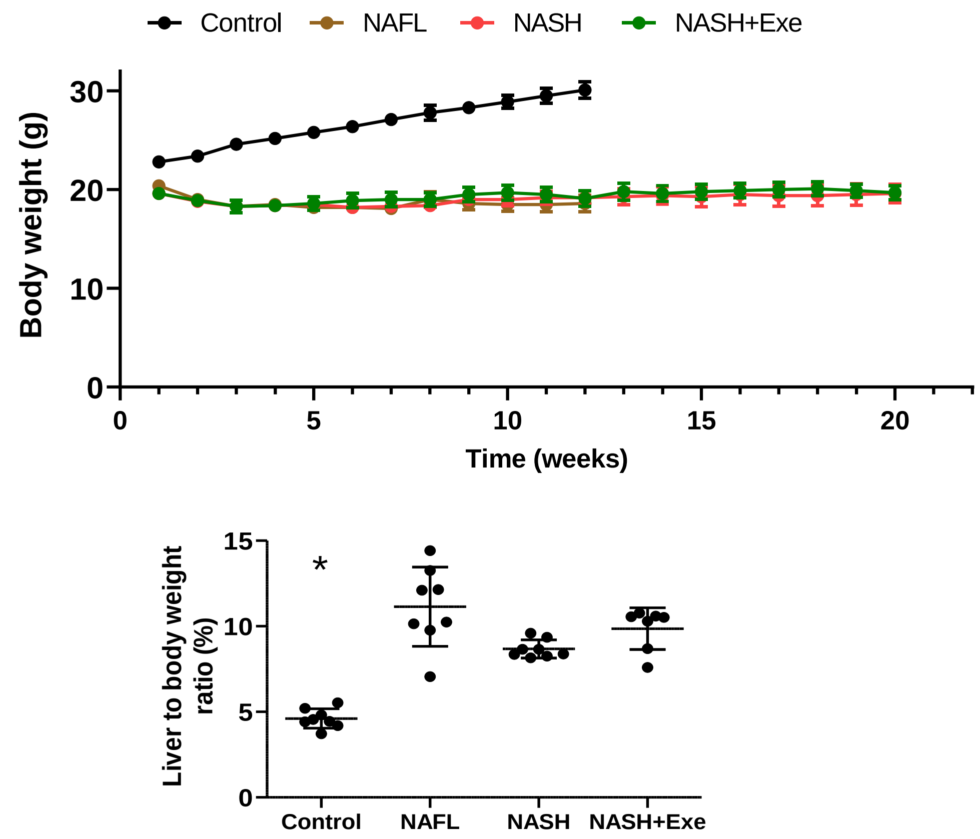

Figure S1: Comparison of body weight and of liver to body weight ratios. C57Bl/6N mice were fed a standard or choline-deficient high-fat (CD-HFD) diet. Mice were randomized to one of four groups: (1) control (n = 11) fed a standard diet for 12 weeks; (2) non-alcoholic fatty liver (NAFL) group (n = 11) fed a CD-HFD diet for 12 weeks; (3) non-alcoholic steatohepatitis (NASH) sedentary group (n = 11) fed a CD-HFD for 20 weeks; (4) NASH + exercise (EXE) group (n = 11) fed a CD-HFD for 20 weeks but with treadmill running at 12.5 m/min imposed from weeks 12 to 20. Body weights were recorded weekly after 7 days of habituation to the diet (upper panel). Tissues were harvested after 12 weeks (control, NAFL) or 20 weeks (NASH sedentary, NASH+ EXE) (lower panel). The liver/body weight ratio was increased in all groups fed the CD-HFD. ($p<0.05$, One way ANOVA).

Figure S2. Unabridged western blot images

Figure 5D

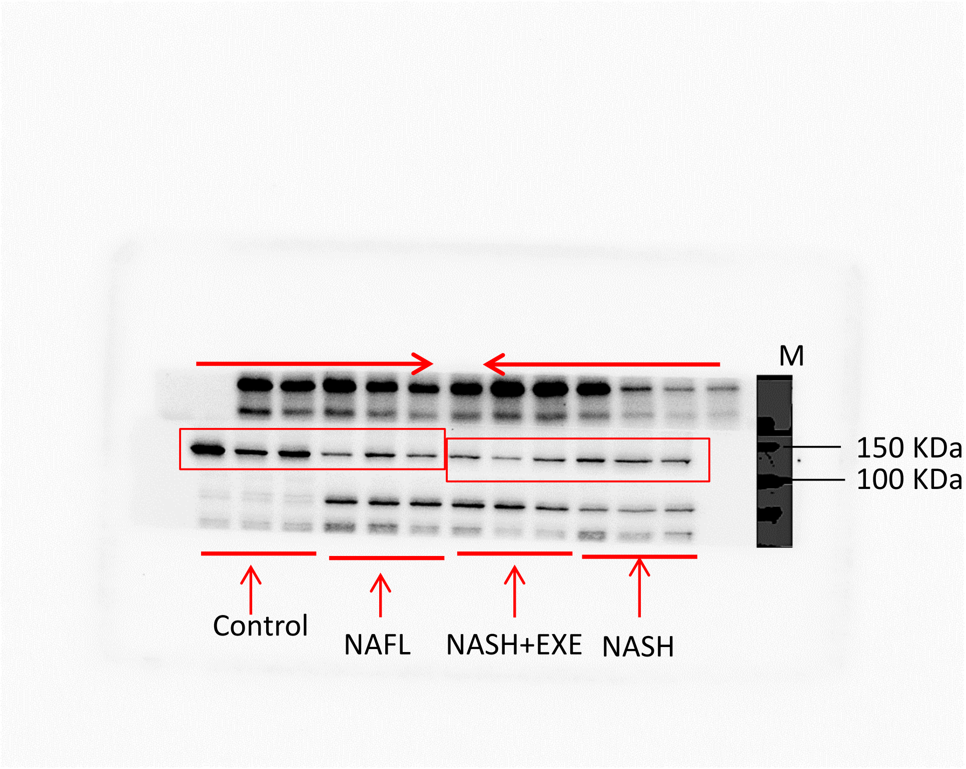

P-ATPCL (predicted MW 125 KDa)

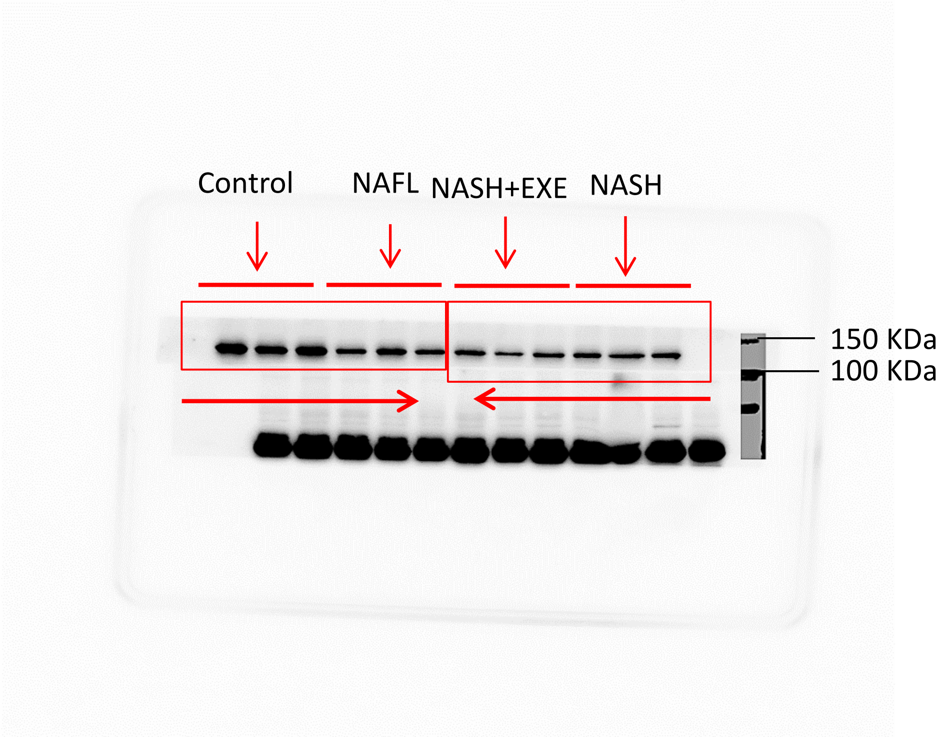

ATPCL (predicted MW 125 KDa)

Figure 5D continued

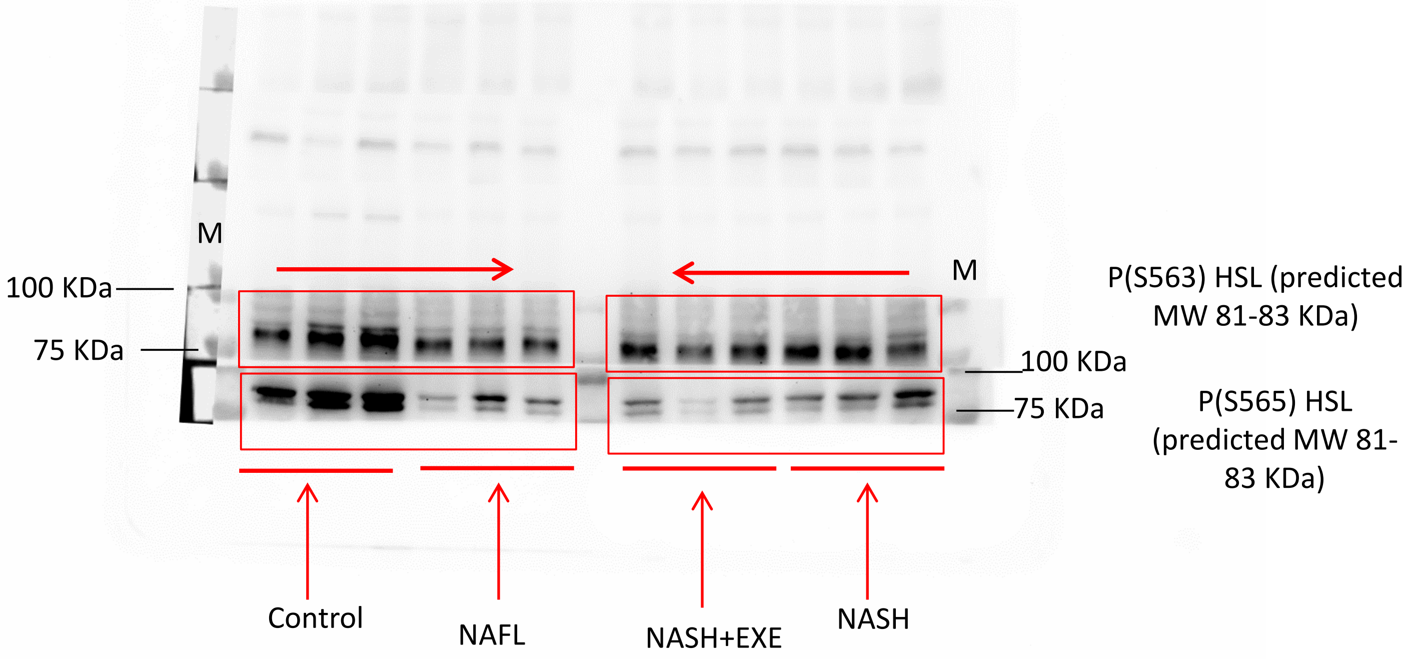

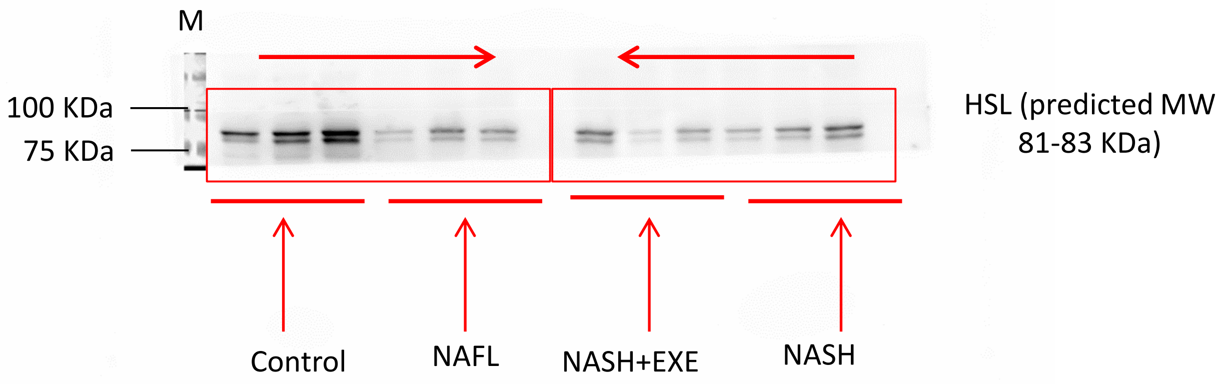

Figure 5D continued

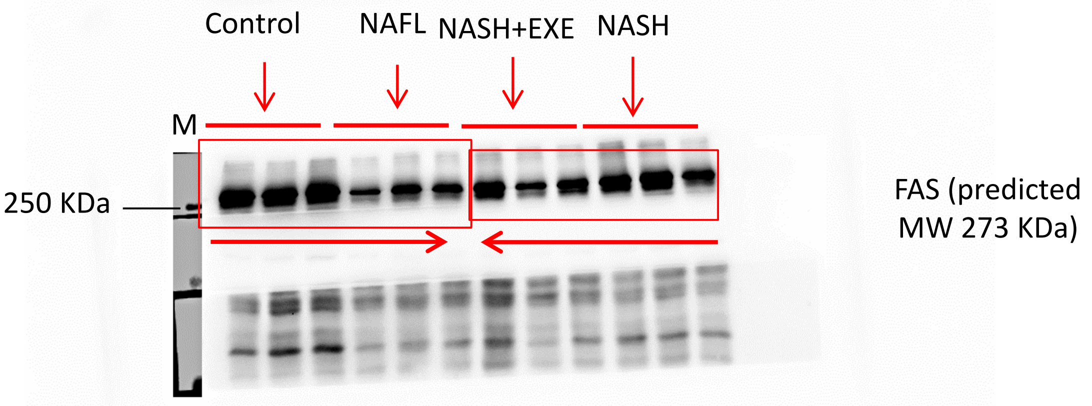

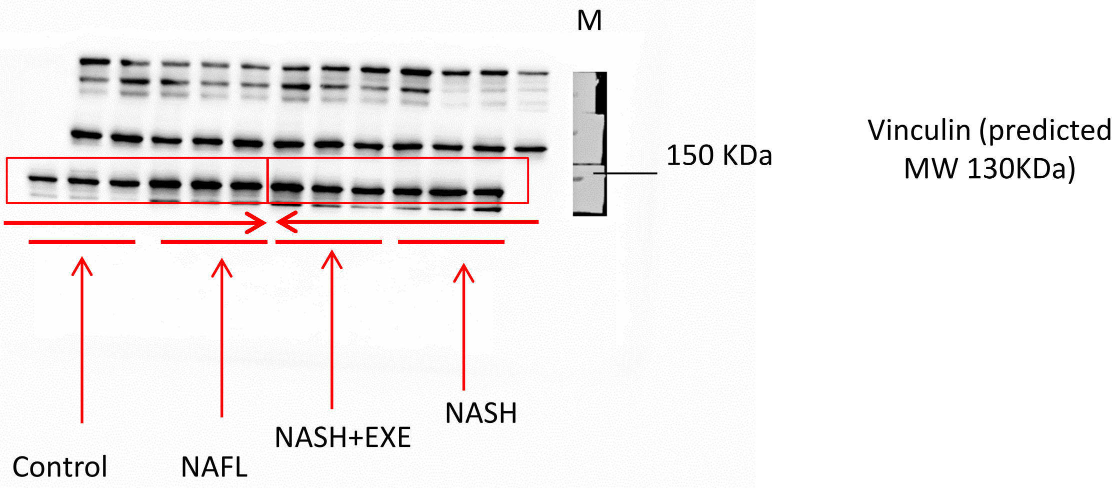

Figure 5D continued

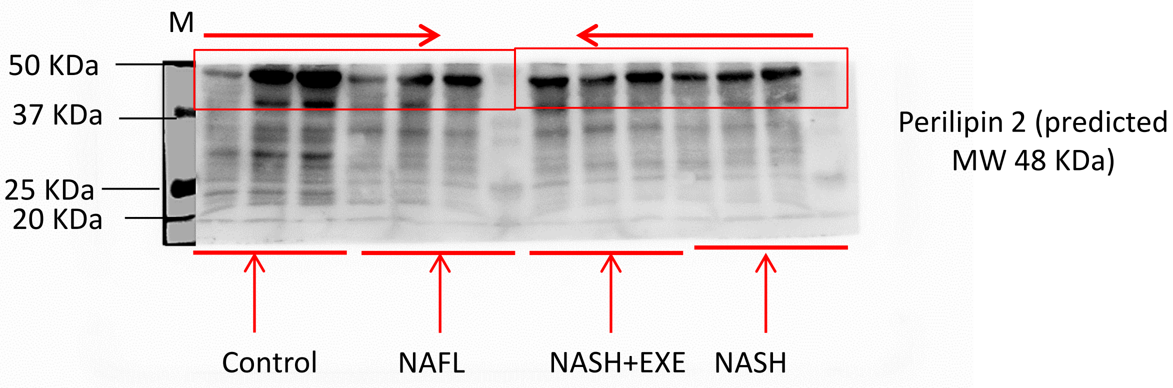

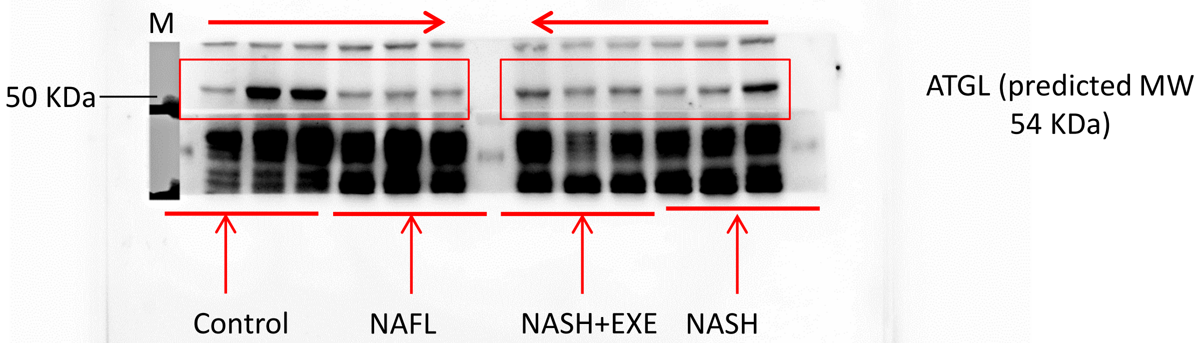

Figure 5D continued

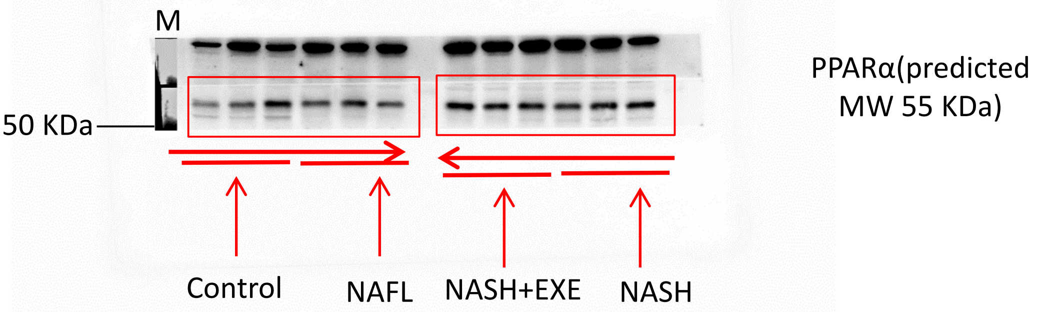

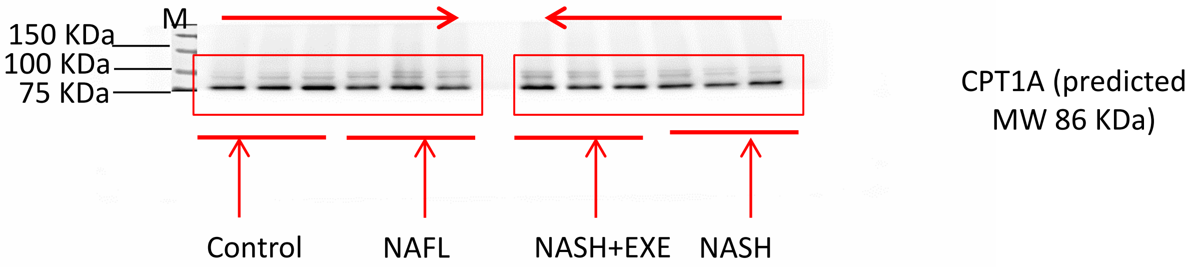

Figure 5D continued

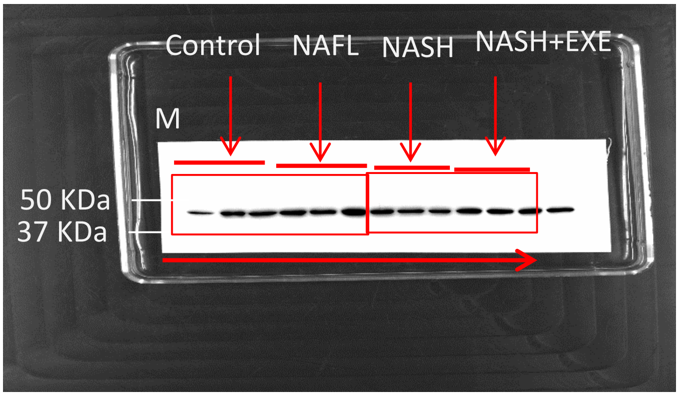

MCAD (predicted MW 47 KDa)

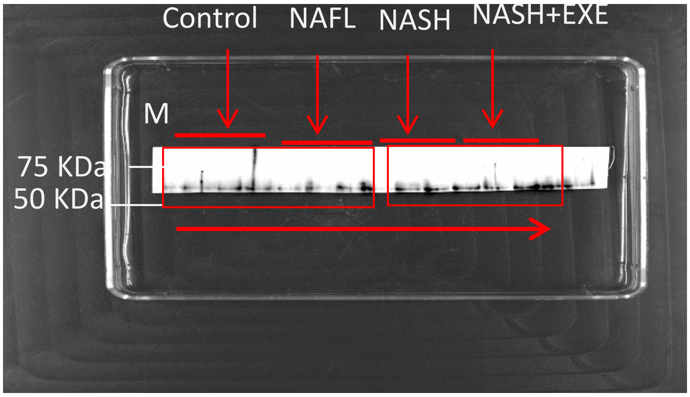

DGAT1 (predicted MW 60 KDa)

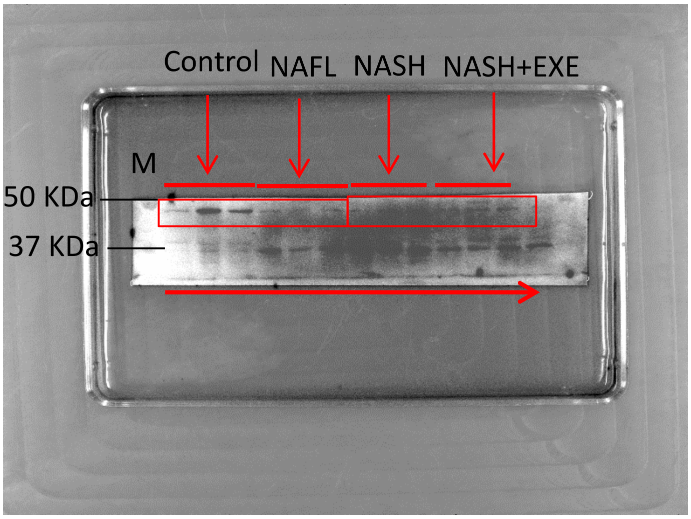

DGAT2 (predicted MW ~47 KDa)

Figure 7

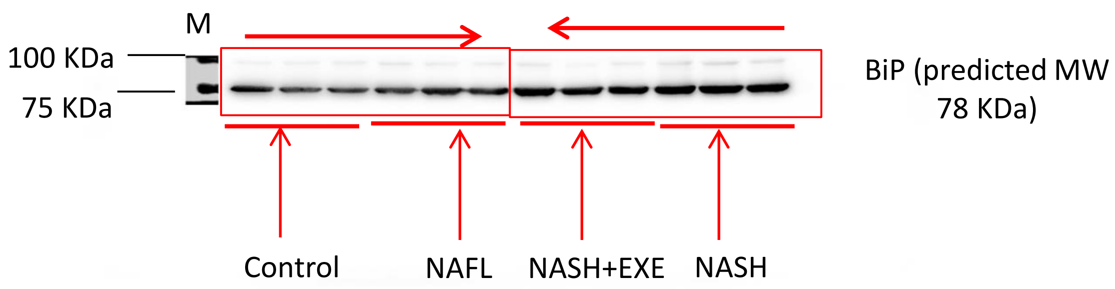
M
100 KDa
75 KDa
BiP (predicted MW 78 KDa)
Control
NAFL
NASH+EXE
NASH

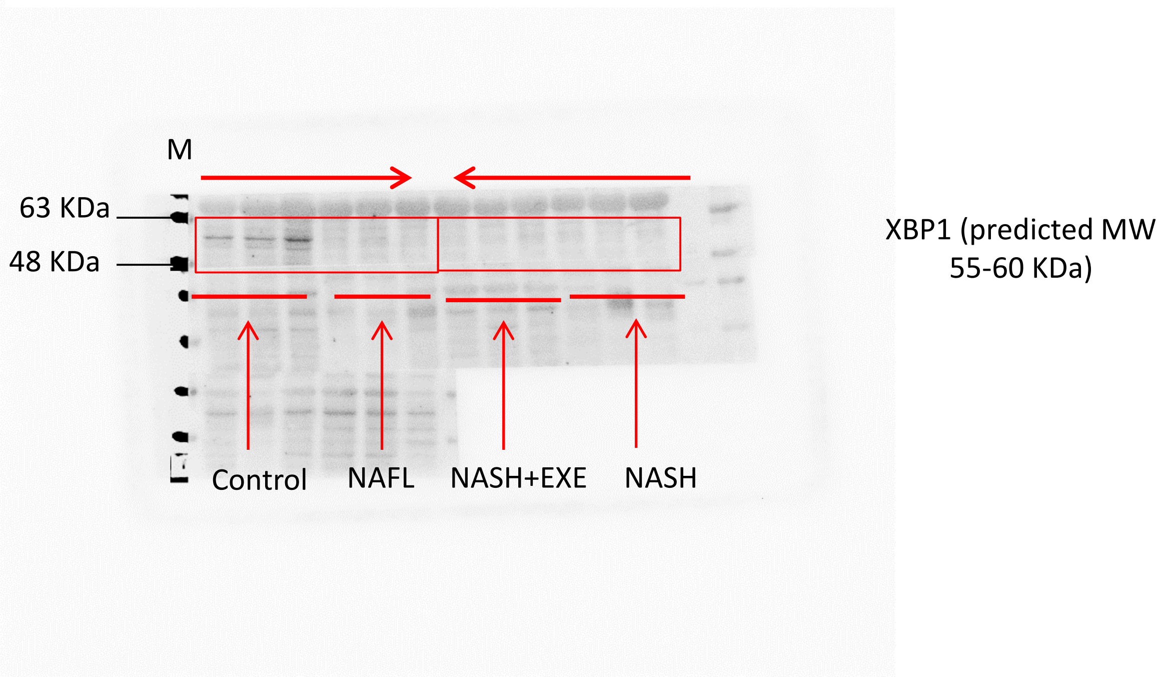
M
63 KDa
48 KDa
XBP1 (predicted MW 55-60 KDa)
Control
NAFL
NASH+EXE
NASH

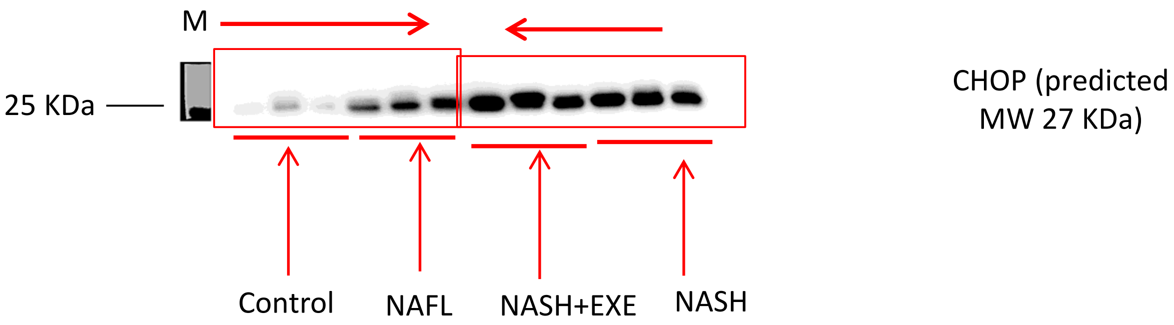
M
25 KDa
CHOP (predicted MW 27 KDa)
Control
NAFL
NASH+EXE
NASH

Figure 7 continued

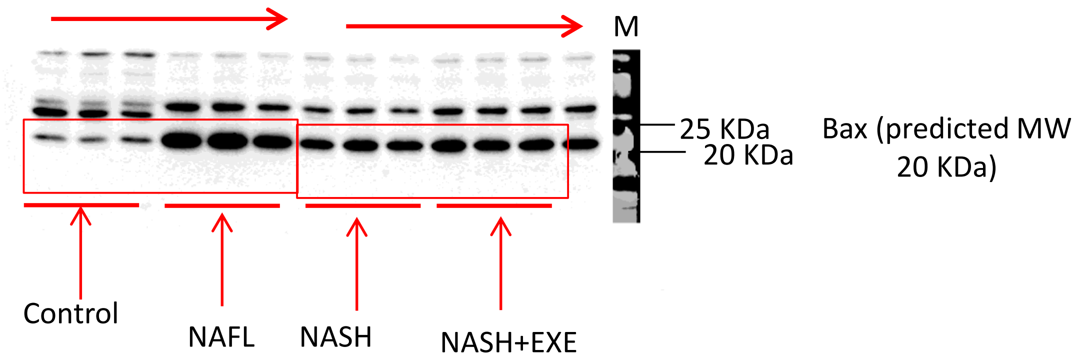

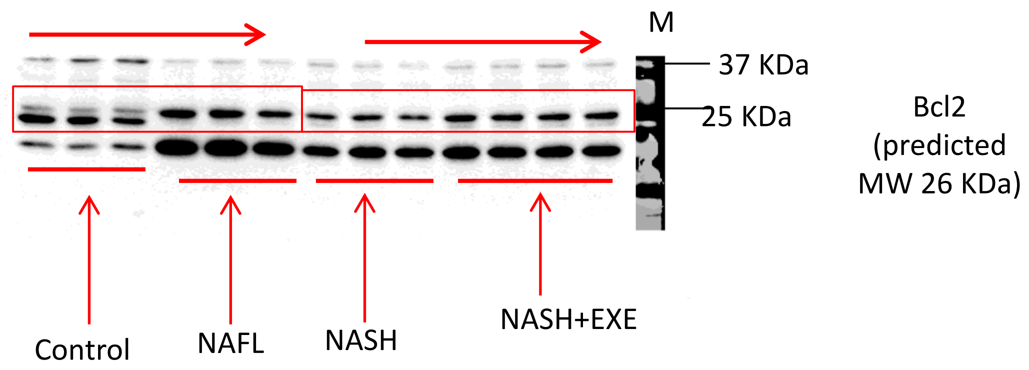

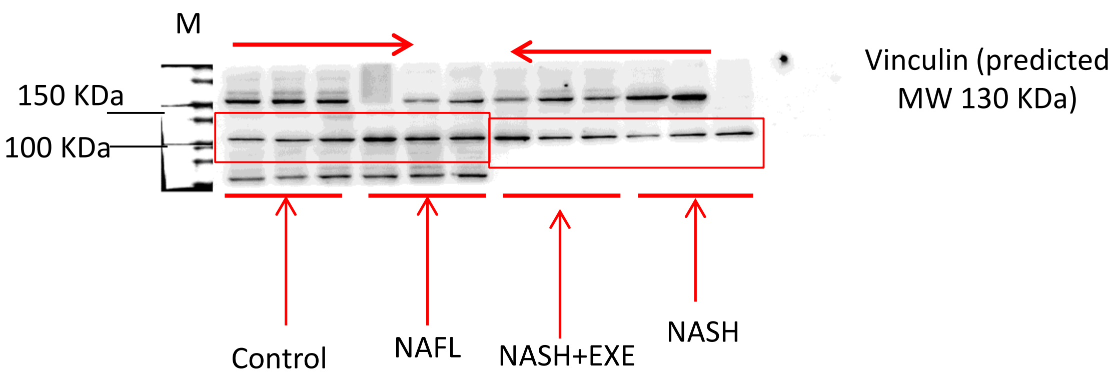

Figure 8

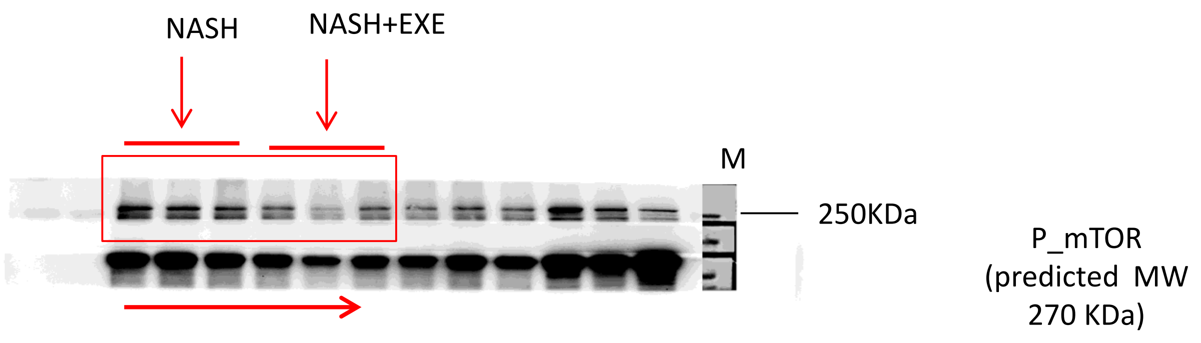
NASH
NASH+EXE
M
250KDa
P_mTOR (predicted MW 270 KDa)

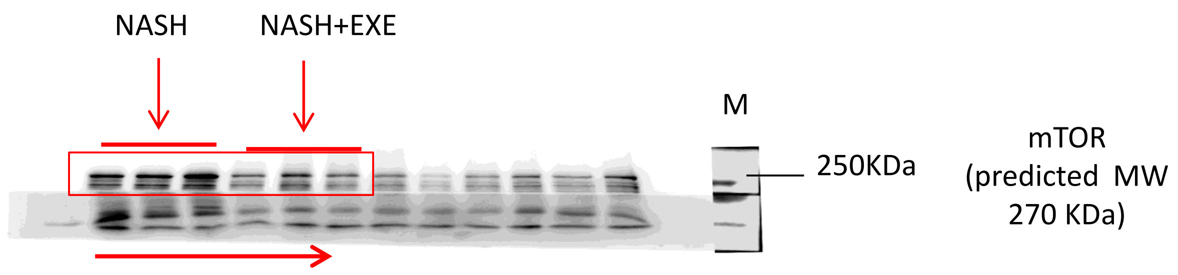
NASH
NASH+EXE
M
250KDa
mTOR (predicted MW 270 KDa)

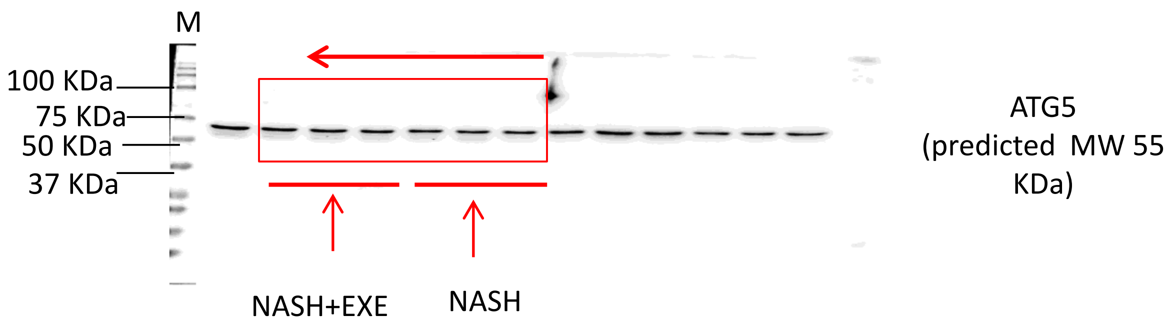
M
100 KDa
75 KDa
50 KDa
37 KDa
NASH+EXE
NASH
ATG5 (predicted MW 55 KDa)

Figure 8 continued

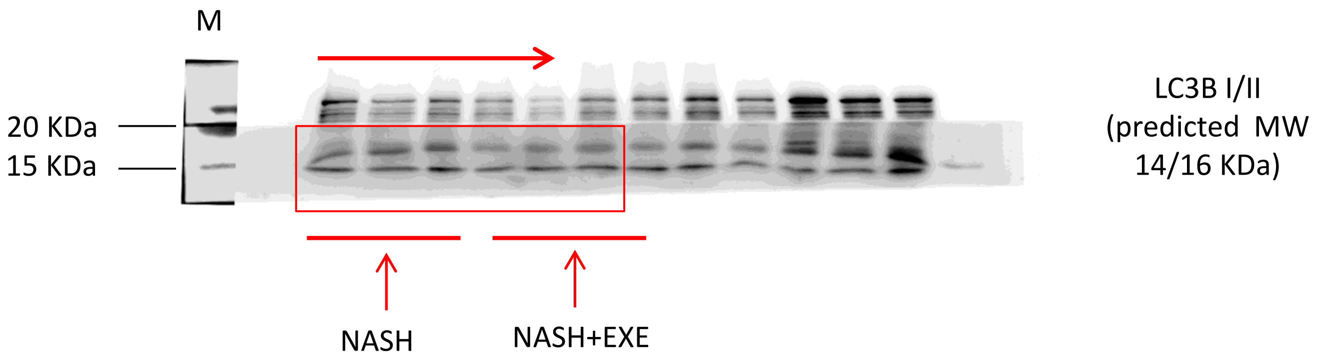

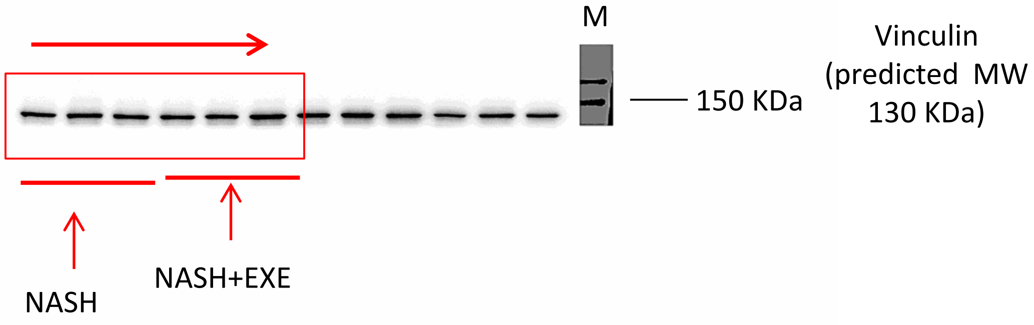

Figure 8 continued

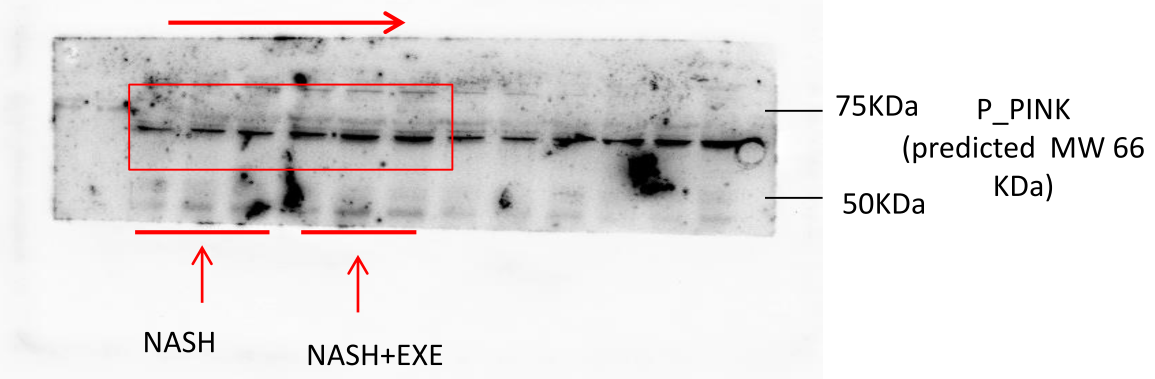

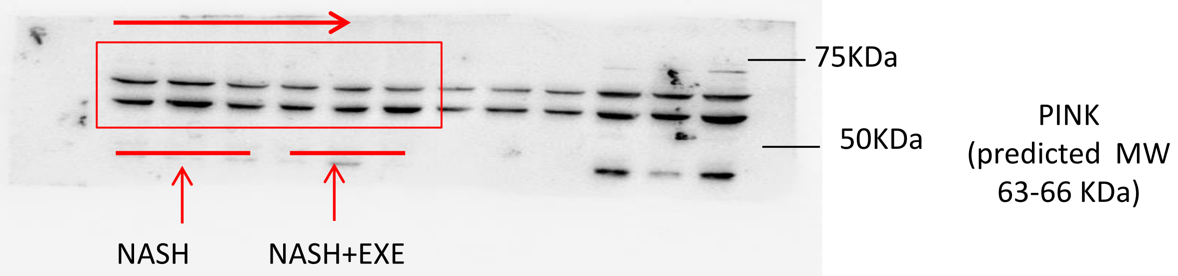

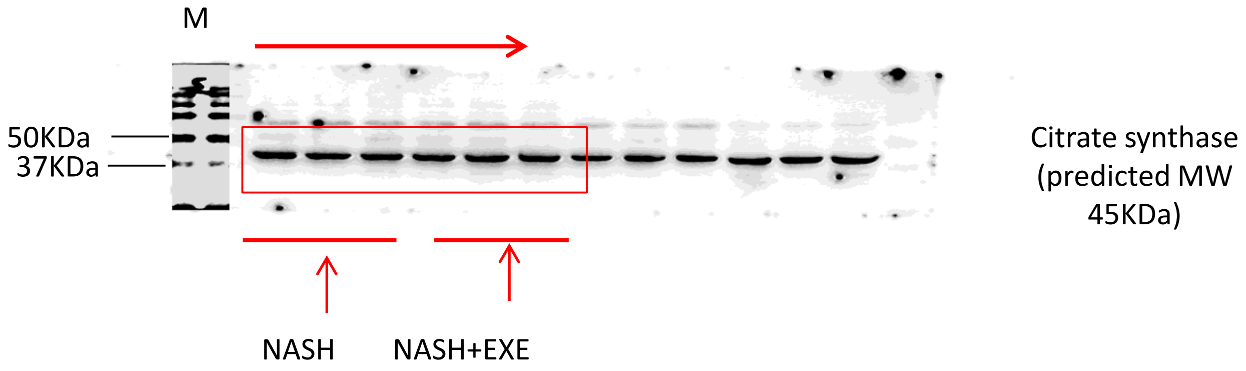

Figure 9C

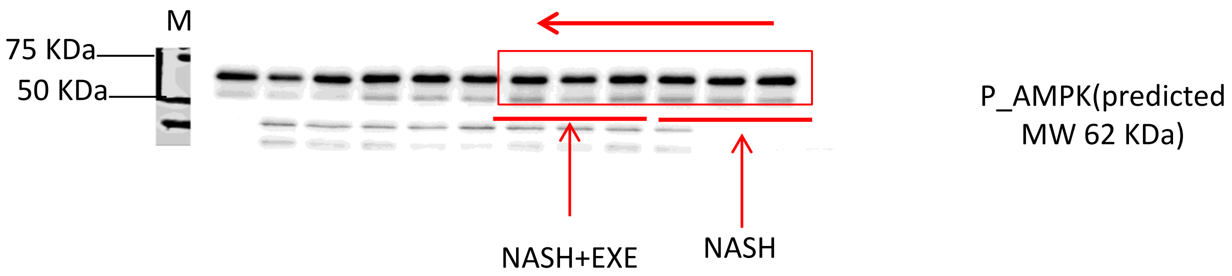
M
75 KDa
50 KDa
NASH+EXE
NASH
P_AMPK(predicted MW 62 KDa)

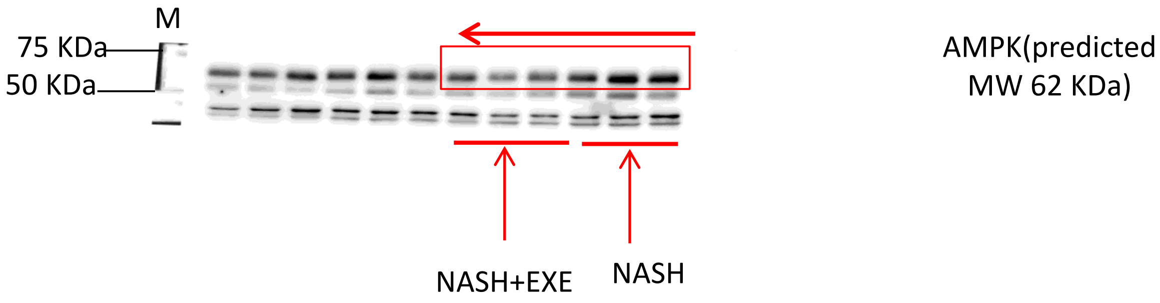
M
75 KDa
50 KDa
NASH+EXE
NASH
AMPK(predicted MW 62 KDa)

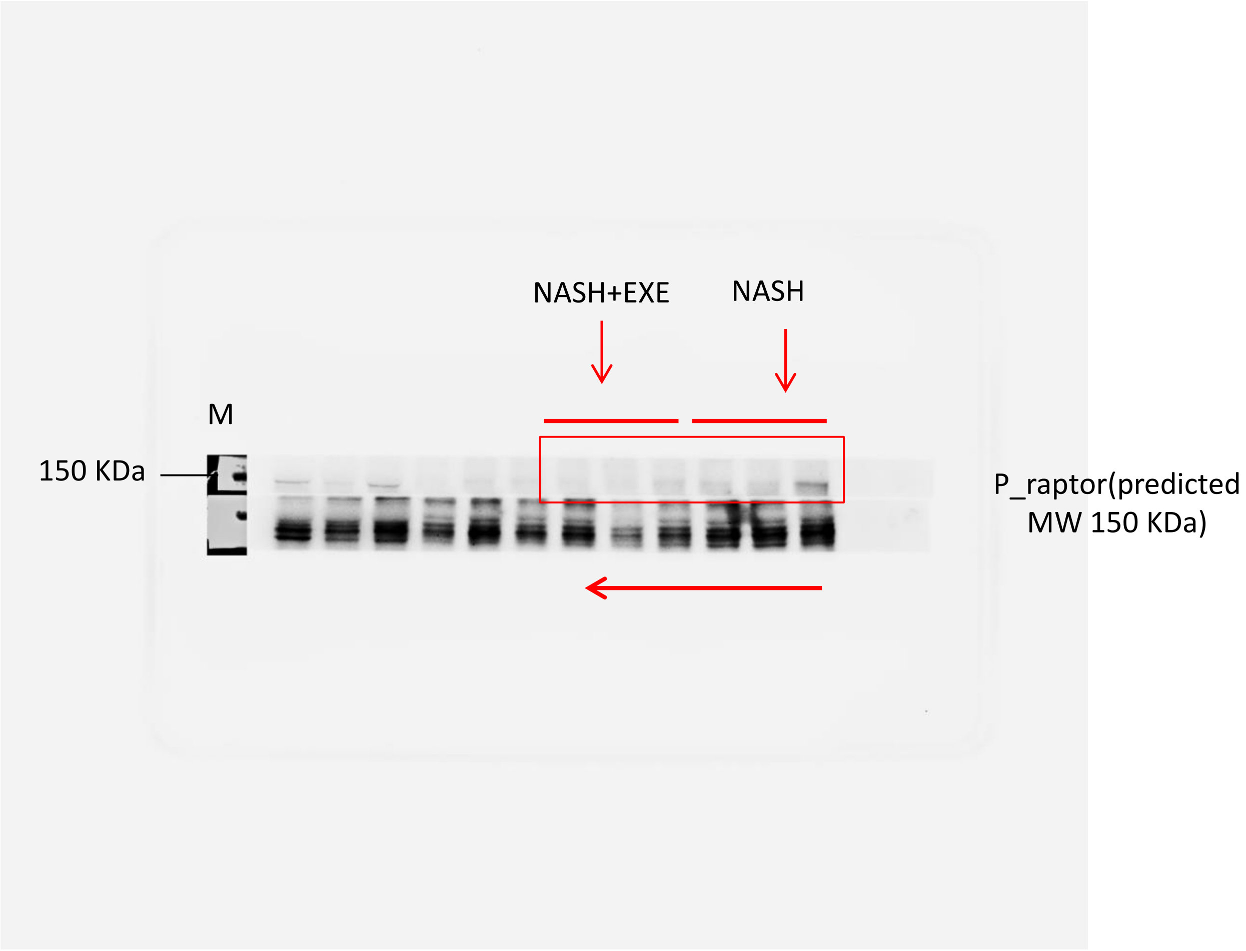
NASH+EXE
NASH
M
150 KDa
P_raptor(predicted MW 150 KDa)

Figure 9C continued

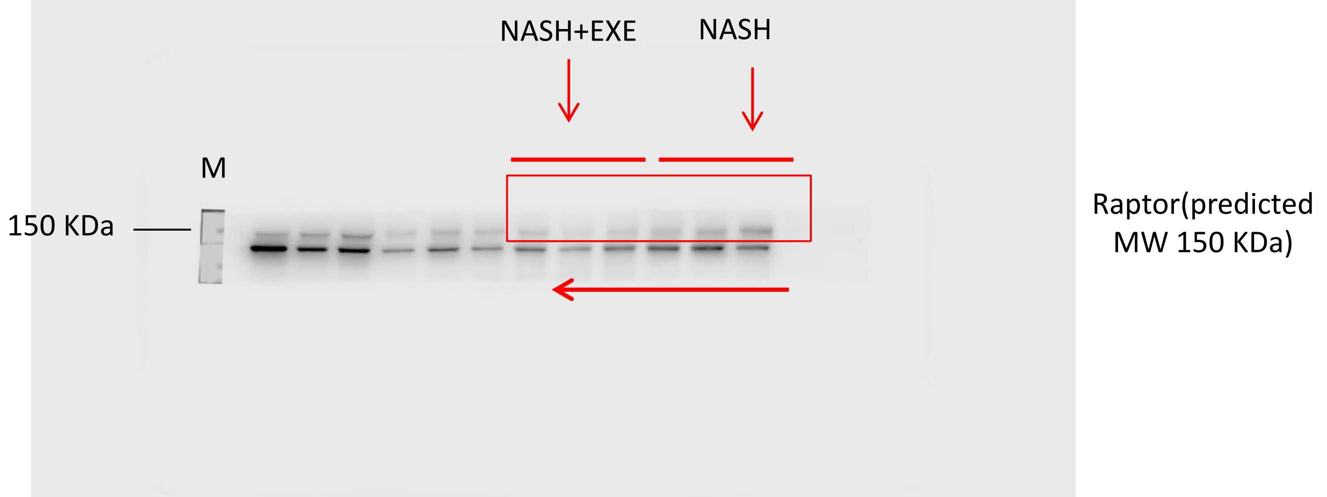

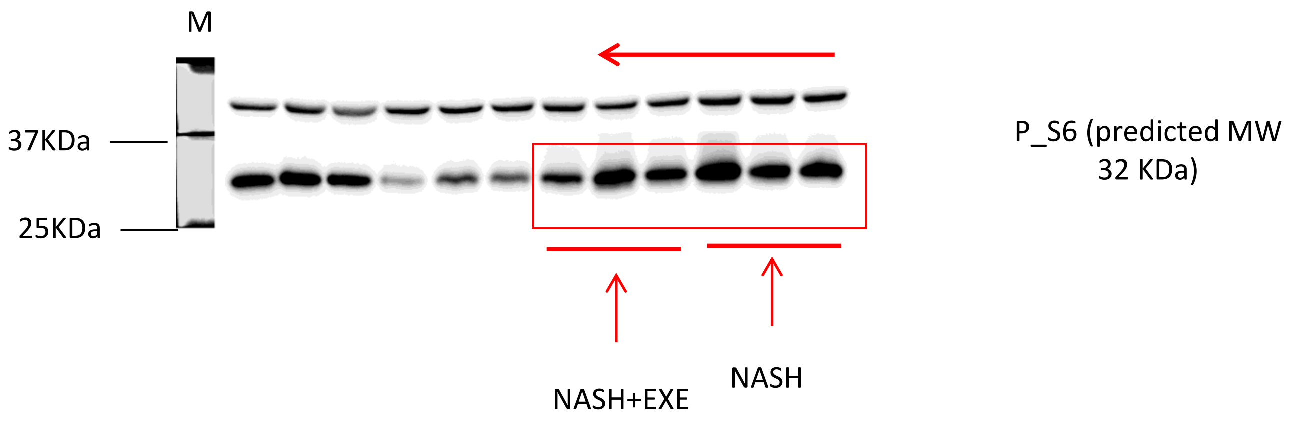

Figure 9C continued

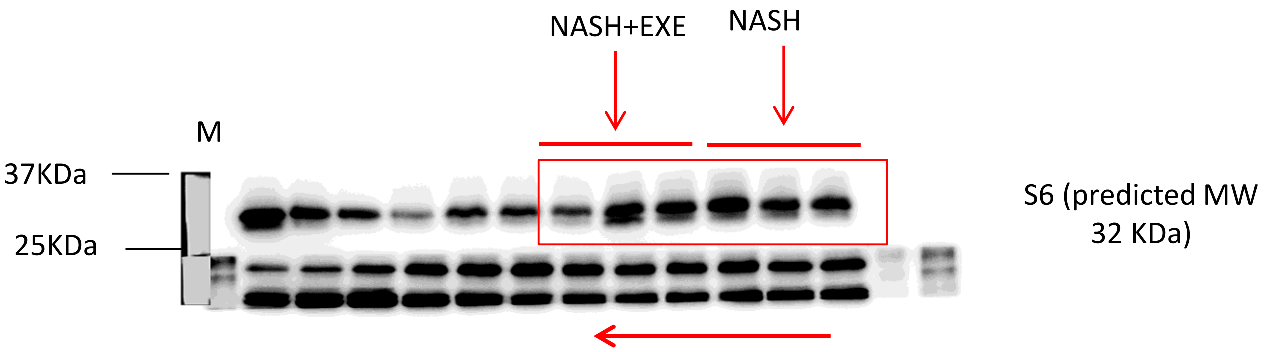

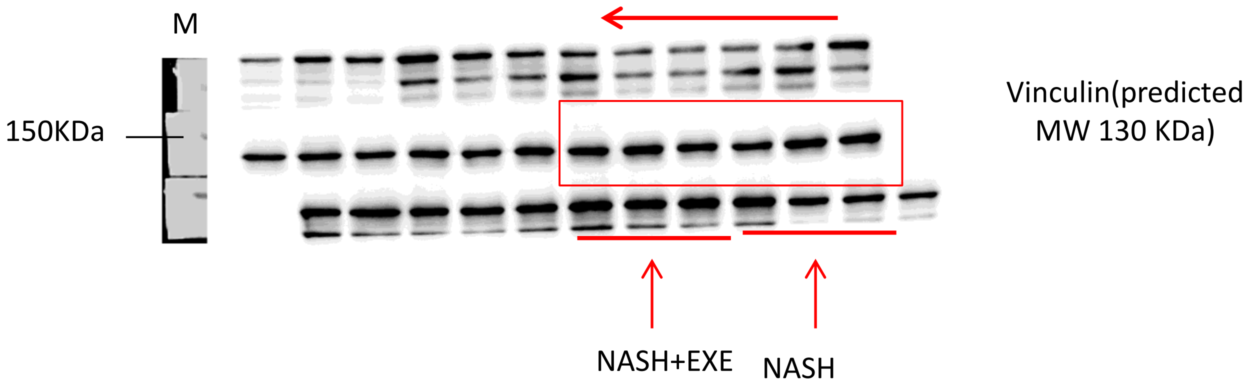

Figure 10 D

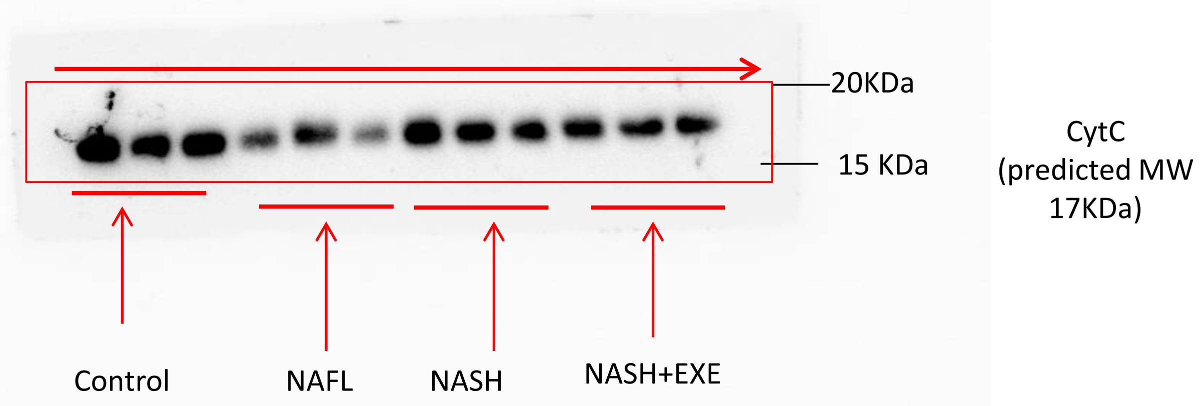

20KDa
15 KDa
CytC (predicted MW 17KDa)
Control
NAFL
NASH
NASH+EXE

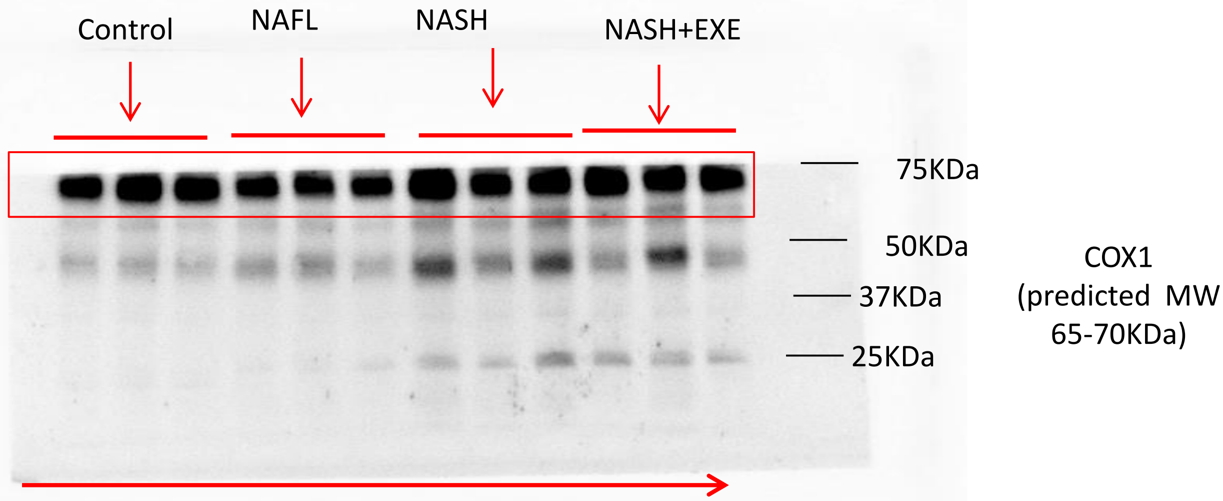

Control
NAFL
NASH
NASH+EXE
75KDa
50KDa
37KDa
25KDa
COX1 (predicted MW 65-70KDa)

Figure 10 D continued

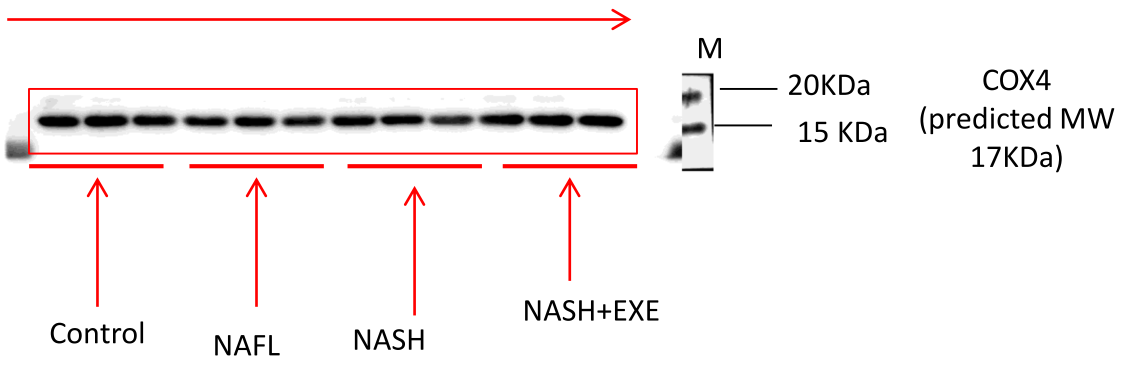

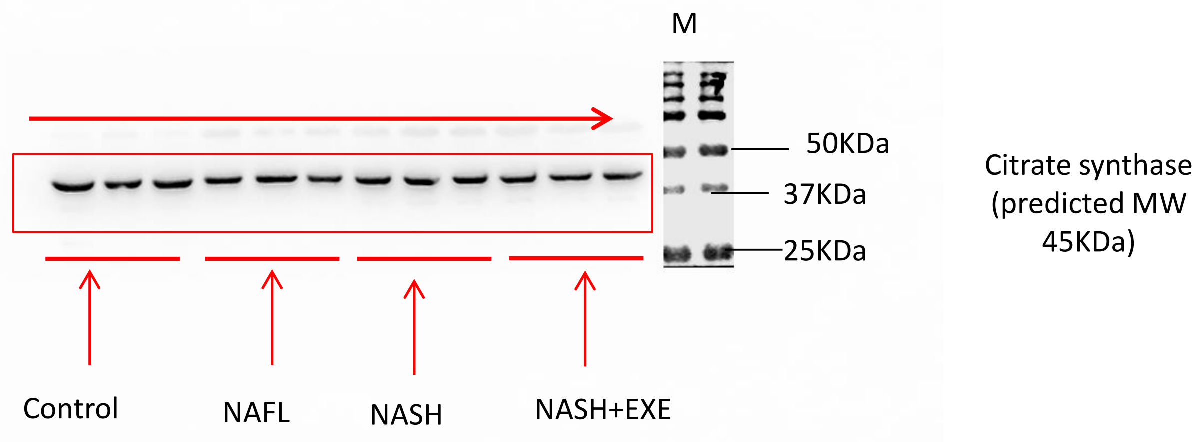

Figure 10 E

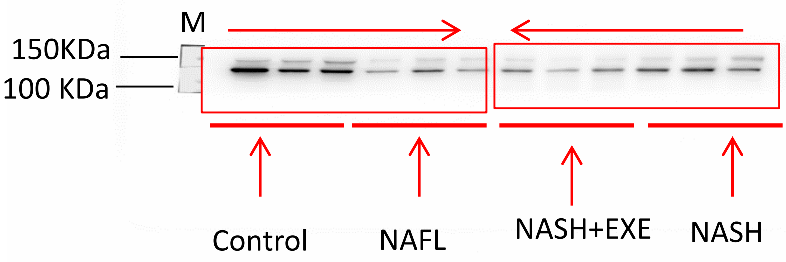

PGC1α (predicted MW 113Da)

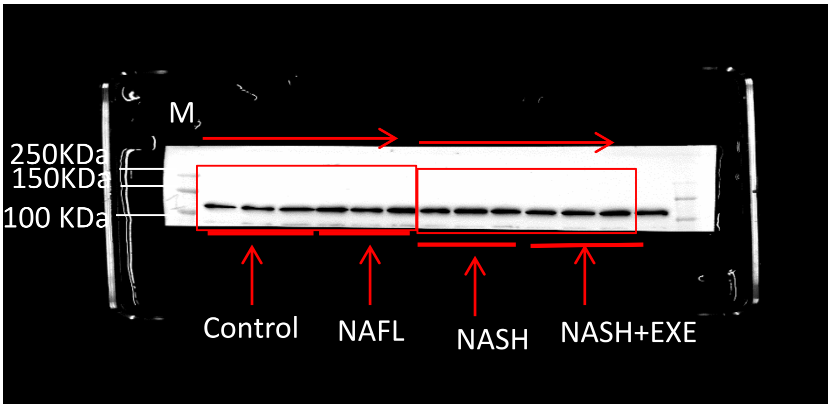

PGC1β (predicted MW 113Da)

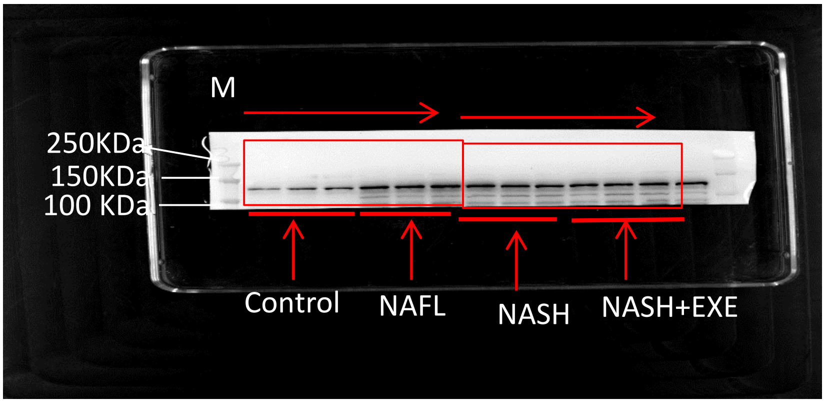

Vinculin (predicted MW 130KDa)

Figure 11B

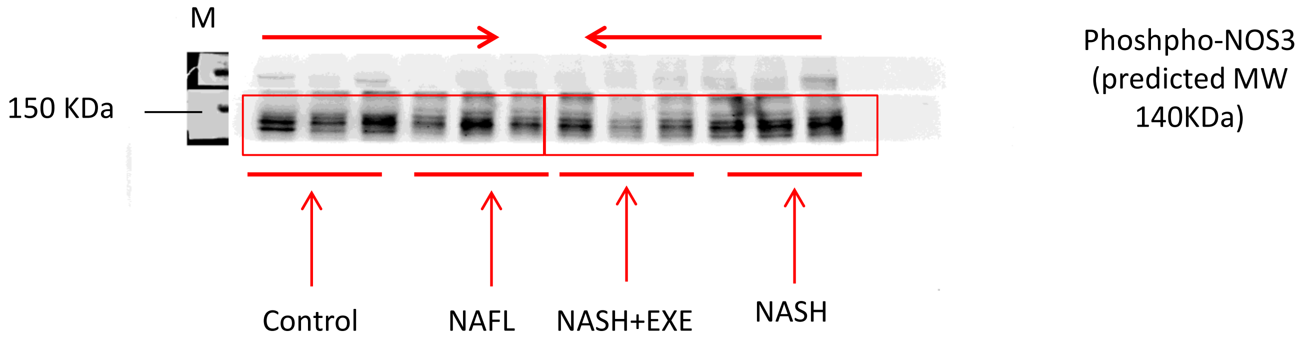

M
150 KDa
Phoshpho-NOS3 (predicted MW 140KDa)
Control
NAFL
NASH+EXE
NASH

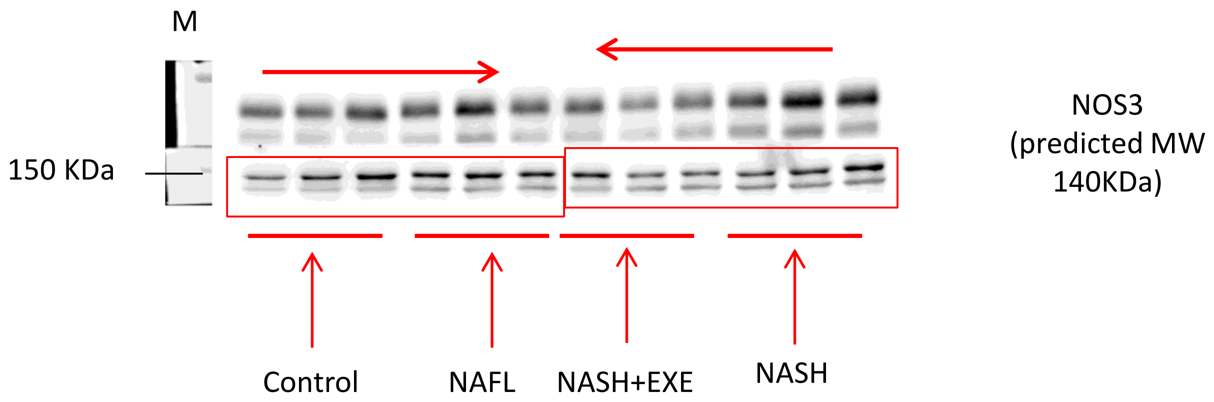

M
150 KDa
NOS3 (predicted MW 140KDa)
Control
NAFL
NASH+EXE
NASH

Figure 11B continued

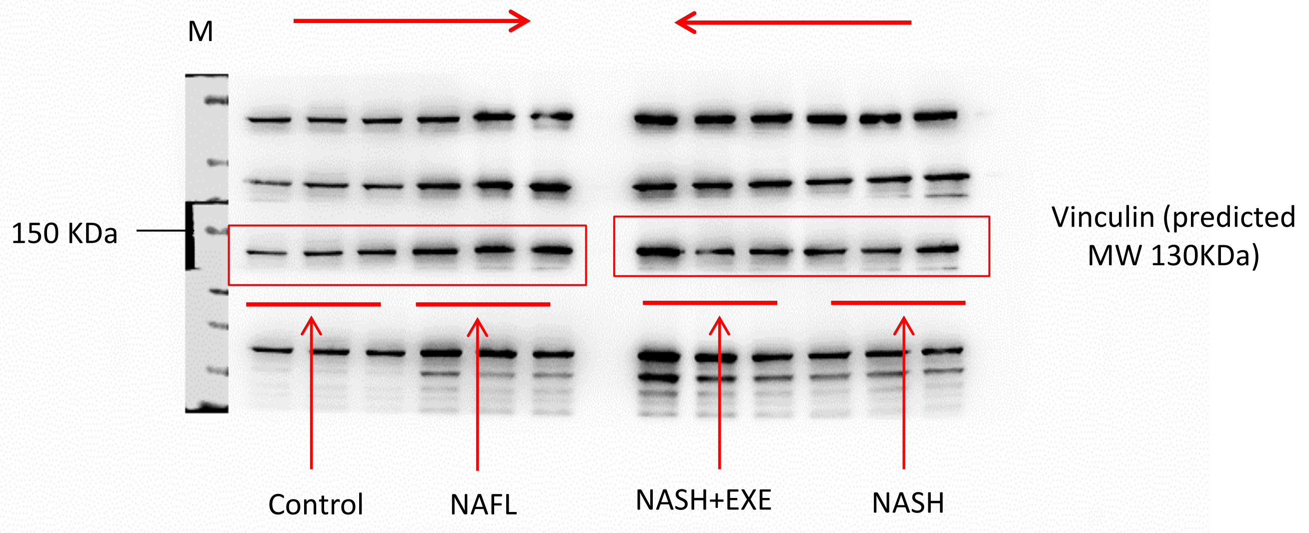

Figure 12

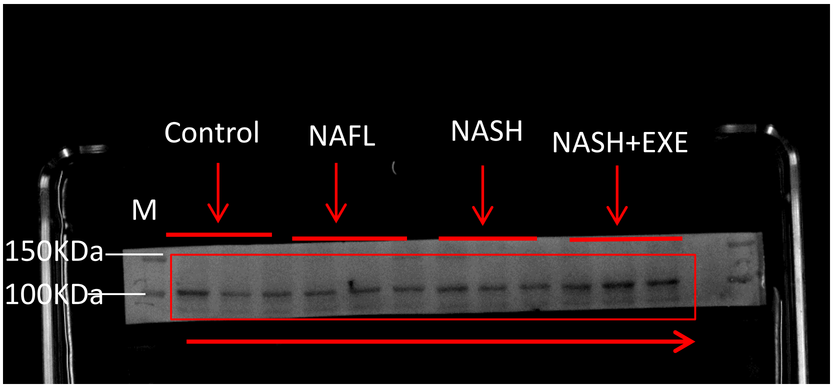

PGC1α
(predicted MW
113Da)

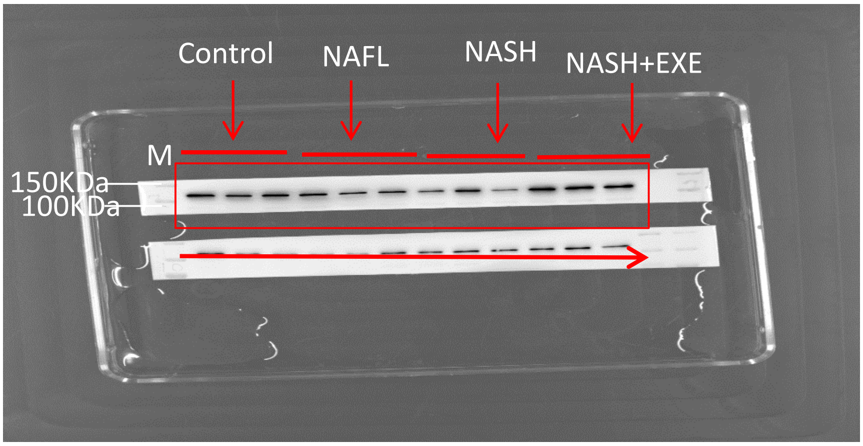

PGC1β
(predicted MW
113Da)

Figure 12 continued

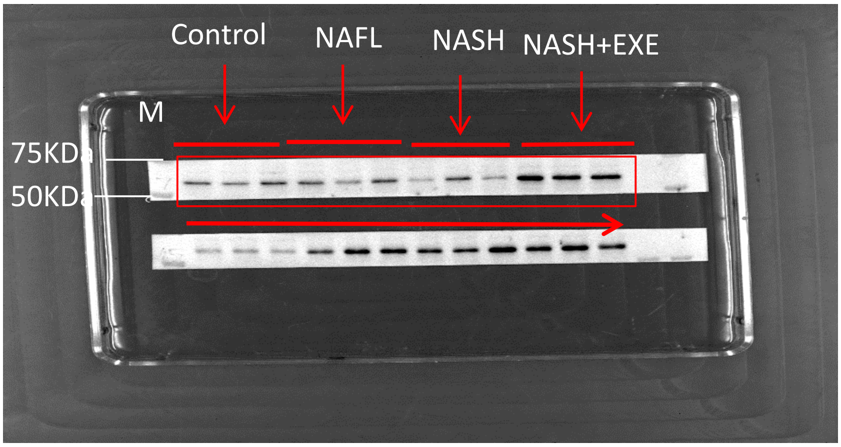

Phospho Akt
(predicted MW
60Da)

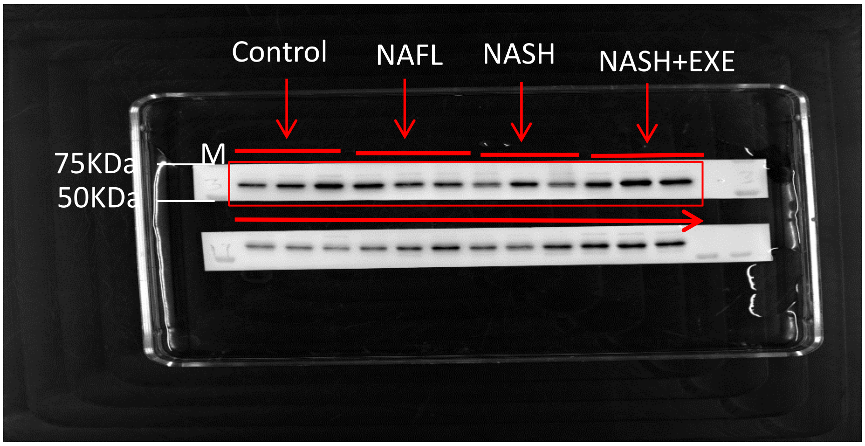

Akt
(predicted MW
60Da)

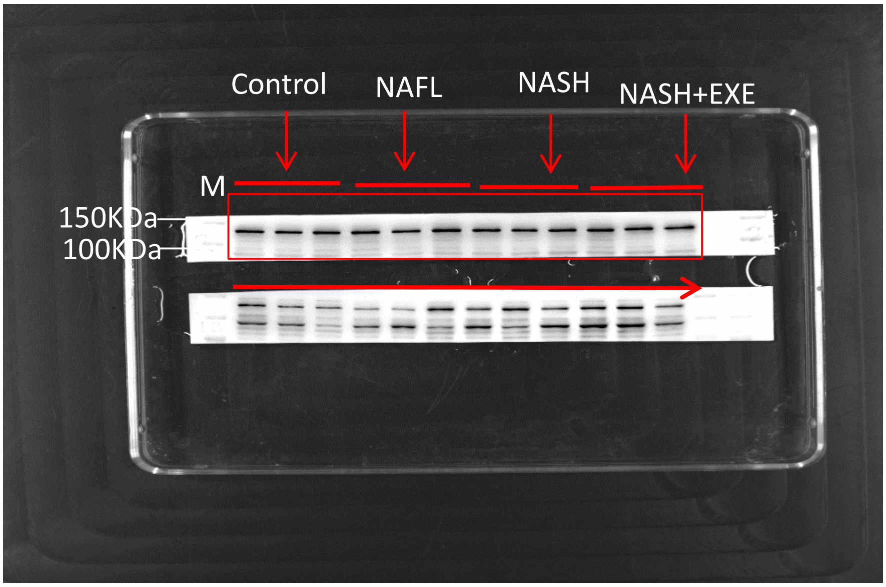

Vinculin
(predicted MW
130KDa)

Table S1

Composition of standard and choline-deficient high-fat diets		
**Ingredient**	**Standard diet (E15668-04) %**	**CD-HFD (E15673-94) %**
Corn starch, pre-gelatinated	40.00	10.00
Maltodextrin	10.00	6.00
Sucrose	18.29	22.00
Cellulose, microcrystalline	5.00	4.95
Amino acid mixture	***14.53***	***14.37***
Vitamin premix AIN w/o choline	1.00	1.00
Mineral & trace element premix	3.50	3.50
Choline Cl (50%)	0.25	-
Sodium bicarbonate	0.43	0.43
Cholesterol	-	2.10
Coconut oil, hydrogenated	-	-
Corn oil	5.00	-
Pork lard	-	34.60
Soybean oil	2.00	1.00
Composition of the amino acid mixture		
L-Lysine HCl	0.91	0.91
L-Methionine	0.40	0.17
L-Cystine	0.30	0.37
L-Threonine	0.46	0.46
**Energy contribution of diet components**		
Energy (Atwater) MJ/Kg	16.60	22.50
Kcal% Protein	12.00	9.00
Kcal% Fat	16.00	60.00
Kcal% Carbohydrates	72.00	31.00

The diets and their compositions were provided by Ssniff Spezialdiäten GmbH, Germany.

Table S2			
**S.No**	**Reagent**	**Source**	**Identifier**
1	Rabbit polyclonal anti-phospho ATP citrate lyase	Cell Signaling Technology	Cat #4331
2	Rabbit polyclonal anti-ATP citrate lyase	Cell Signaling Technology	Cat #4332
3	Rabbit monoclonal anti-fatty acid synthase	Cell Signaling Technology	Cat #3180
4	Rabbit polyclonal anti-Phospho (S563)-HSL	Cell Signaling Technology	Cat #4139
5	Rabbit polyclonal anti-Phospho (S565)-HSL	Cell Signaling Technology	Cat #4137
6	Rabbit polyclonal anti-HSL	Cell Signaling Technology	Cat #4107
7	Rabbit polyclonal anti-perilipin 2	Proteintech	Cat #15294-1-AP
8	Rabbit monoclonal anti-ATGL	Cell Signaling Technology	Cat #2439
9	Rabbit polyclonal anti-PPARα	Santa Cruz Biotechnology	Cat #sc-9000
10	Rabbit polyclonal anti-CPT1A	Proteintech	Cat #15184-1-AP
11	Mouse monoclonal anti-MCAD	Abcam	Cat #ab110296
12	Rabbit polyclonal anti-DGAT1	Abnova	Cat #PAB8674
13	Goat polyclonal anti-DGAT2	Novus Biologicals	Cat #NB100-57851
14	Mouse monoclonal anti-vinculin	Merck Milipore	Cat #CP74
15	Rabbit polyclonal anti-(GRP78) BiP	Thermo Fisher	Cat #PA5-34941
16	Rabbit polyclonal anti-XBP1s	Cell Signaling Technology	Cat #83418
17	Mouse monoclonal anti-CHOP	Cell Signaling Technology	Cat #2895
18	Rabbit polyclonal anti-Bax	Cell Signaling Technology	Cat #2772
19	Rabbit polyclonal anti-Bcl2	Cell Signaling Technology	Cat #2876
20	Rabbit polyclonal anti-phospho mTOR	Cell Signaling Technology	Cat #2971

Table S2 continued			
**S.No**	**Reagent**	**Source**	**Identifier**
21	Rabbit monoclonal anti-mTOR	Cell Signaling Technology	Cat #2983
22	Rabbit monoclonal anti-ATG5	Cell Signaling Technology	Cat #12994
23	Rabbit polyclonal anti-LC3B	Abcam	Cat #ab48394
24	Rabbit polyclonal anti-phospho PINK	Affinity Biosciences	Cat #AF7081
25	Rabbit polyclonal anti-PINK	Cayman Chemical	Cat #10006283
26	Rabbit monoclonal anti-citrate synthase	Cell Signaling Technology	Cat #14309
27	Rabbit polyclonal anti-phospho AMPK	Cell Signaling Technology	Cat #2531
28	Rabbit polyclonal anti-AMPK	Cell Signaling Technology	Cat #2532
29	Rabbit polyclonal anti-phospho raptor	Cell Signaling Technology	Cat #2083
30	Rabbit monoclonal anti-raptor	Cell Signaling Technology	Cat #2280
31	Rabbit polyclonal anti-phospho S6	Cell Signaling Technology	Cat #2215
32	Rabbit monoclonal anti-S6	Cell Signaling Technology	Cat #2217
33	Rabbit polyclonal anti-cytochrome C	Cell Signaling Technology	Cat #4272
34	Rabbit polyclonal anti-(MTCO1)COX1	Novus Biologicals	Cat #NBP2-29949
35	Rabbit polyclonal anti-COXIV	Proteintech	Cat #11242-1-AP
36	Mouse monoclonal anti-PGC1α	Merck Milipore	Cat #ST1202
37	Rabbit monoclonal anti-PGC1β	Abcam	Cat #ab176328
38	Rabbit polyclonal anti-phospho NOS3	Cell Signaling Technology	Cat #9571
39	Mouse monoclonal anti-NOS3	BD biosciences	Cat #610297
40	Rabbit polyclonal anti-phospho (S473) AKT	Cell Signaling Technology	Cat #9271
41	Rabbit polyclonal anti-AKT	Cell Signaling Technology	Cat #9272

**Table S3**					
**S.No.**	**Assay ID**	**Dye**	**Gene Symbol**	**Gene Name**	**Identifier**
1	Mm99999056_m1	FAM	Ccl2	Chemokine ligand 2 MCP-1	Thermo Fisher Scientific
2	Mm01135198_m1	FAM	Cd36	CD36 antigen	Thermo Fisher Scientific
3	Mm00439560_m1	FAM	IGF1	Insulin-like growth factor 1	Thermo Fisher Scientific
4	Mm00439564_m1	FAM	IGF2	Insulin-like growth factor 2	Thermo Fisher Scientific
5	Mm00449517_m1	FAM	FATP2	Fatty acid transporter, member 2	Thermo Fisher Scientific
6	Mm00447768_m1	FAM	FATP5	Fatty acid transporter, member 5	Thermo Fisher Scientific
7	Mm01178820_m1	FAM	Tgfb1	Transforming growth factor, beta 1	Thermo Fisher Scientific
8	Mm00437762 PL	VIC	B2m	B-microglobulin Primer Limited	Thermo Fisher Scientific
9	Mm00443258_m1	FAM	TNF	Tumor necrosis factor	Thermo Fisher Scientific
10	Mm00446190_m1	FAM	Il6	Interleukin 6	Thermo Fisher Scientific
11	Mm00840165_g1	FAM	FGF21	Fibroblast growth factor 21	Thermo Fisher Scientific
